# Quinolinium-Based Fluorescent Probes for Dynamic pH Monitoring in Aqueous Media at High pH Using Fluorescence Lifetime Imaging

**DOI:** 10.1021/acssensors.3c00316

**Published:** 2023-04-27

**Authors:** Jorrit Bleeker, Aron P. Kahn, Lorenz M. Baumgartner, Ferdinand C. Grozema, David A. Vermaas, Wolter F. Jager

**Affiliations:** Faculty of Applied Sciences, Department of Chemical Engineering, Delft University of Technology, Delft 2629 HZ, The Netherlands

**Keywords:** FLIM, FLIM probe, fluorescence lifetime, fluorescent pH probe, molecular probe, quinolinium dye, alkaline pH probing

## Abstract

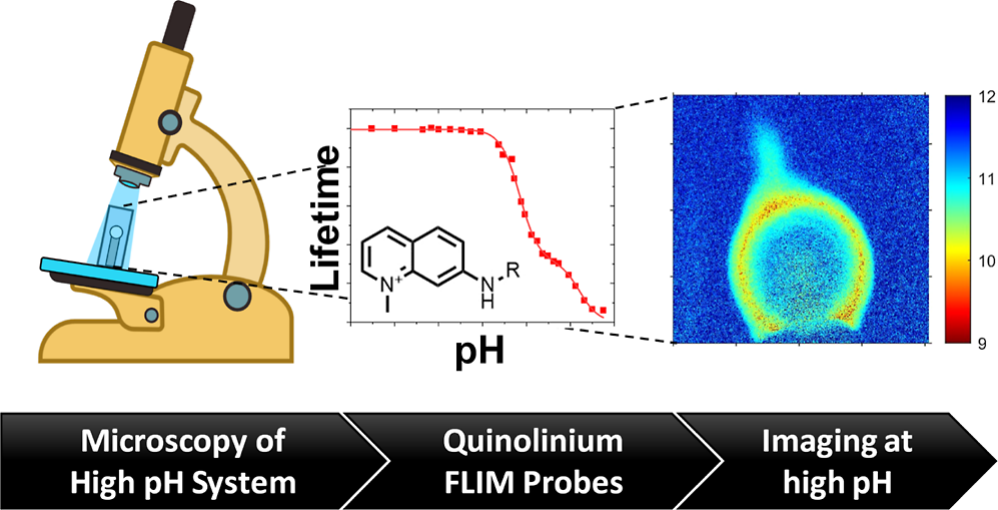

Spatiotemporal pH imaging using fluorescence lifetime imaging microscopy (FLIM) is an excellent technique for investigating dynamic (electro)chemical processes. However, probes that are responsive at high pH values are not available. Here, we describe the development and application of dedicated pH probes based on the 1-methyl-7-amino-quinolinium fluorophore. The high fluorescence lifetime and quantum yield, the high (photo)stability, and the inherent water solubility make the quinolinium fluorophore well suited for the development of FLIM probes. Due to the flexible fluorophore-spacer–receptor architecture, probe lifetimes are tunable in the pH range between 5.5 and 11. An additional fluorescence lifetime response, at tunable pH values between 11 and 13, is achieved by deprotonation of the aromatic amine at the quinolinium core. Probe lifetimes are hardly affected by temperature and the presence of most inorganic ions, thus making FLIM imaging highly reliable and convenient. At 0.1 mM probe concentrations, imaging at rates of 3 images per second, at a resolution of 4 μm, while measuring pH values up to 12 is achieved. This enables the pH imaging of dynamic electrochemical processes involving chemical reactions and mass transport.

Imaging chemical and biological systems can give insights into the local mass transport and reaction kinetics. Fluorescent molecular probes are excellent materials for characterizing these dynamic processes.^1,2^ Characteristics of the medium that are probed may be physical in nature, such as measurements of temperature,^3−6^ pressure,^7^ mechanical stress,^8^ solvent mobility,^9−11^ or solvent polarity.^12^ Alternatively, the chemical composition of the medium, such as pH,^13−16^ the concentration of ions,^17,18^ or more complex chemical species,^19−21^ can be monitored. A distinct advantage of fluorescent probes is their inherently low detection threshold. Fluorescence can be detected at very low probe concentrations, in principle down to the single molecule, but routinely in the micromolar range (10^–6^ M). Because fluorescent probes can be localized by microscopic techniques and fluorescence can be monitored in real time, the use of fluorescent probes enables *spatiotemporal* probing.

In order to detect chemical species, the fluorescence of probe molecules should be influenced by interaction with analytes. In most cases, this probe–analyte interaction is a reversible binding event. The most convenient and flexible probe architecture is the modular fluorophore–spacer–receptor configuration,^22^ in which a receptor is attached to a fluorophore by a flexible spacer, see Figure 1. Upon binding the analyte to the receptor, the fluorophore emission is altered and the most commonly encountered fluorescence response is a change in emission intensity. In most cases, intensity changes are induced by changing the nonradiative decay rate of the excited state *k*_nr_, as described by eqs 1 and 2. In these equations, *k*_F_ and *k*_nr_ are the rate constants for fluorescence and nonradiative decay, respectively, and Φ_F_ and τ_F_ are the quantum yield and the lifetime of fluorescence. Eqs 1 and 2 clearly express that if the binding event only changes *k*_nr_, which is generally the case for fluorophore–spacer–receptor probes, Φ_F_ and τ_F_ will be proportional.
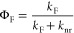
1
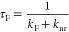
2

**Figure 1:**
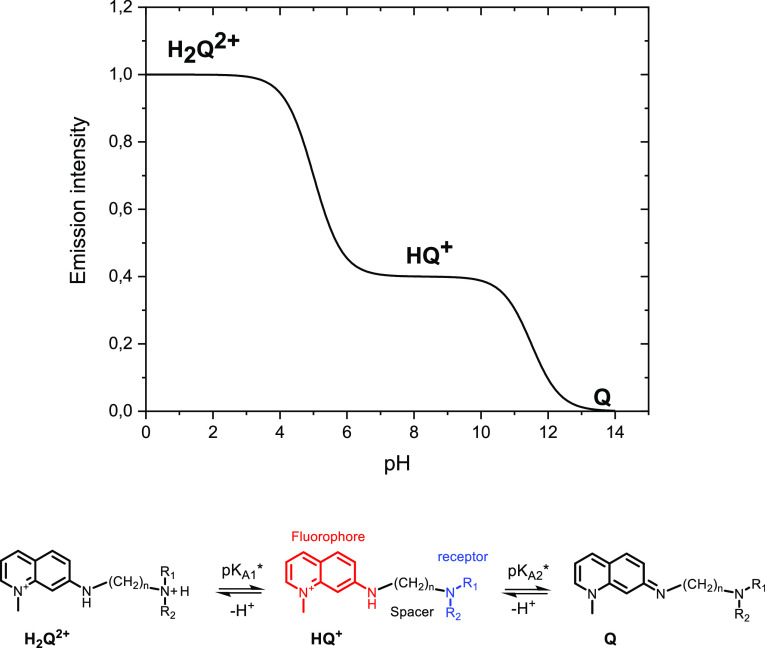
General emission intensity vs pH behavior of quinolinium probes. Values used for constructing this graph are p*K*_A1_^*^ = 5, FE = 2, and p*K*_A2_^*^ = 11.5.

Although an altered fluorescence intensity provides information about the local analyte concentration, this is not the most desired probe response because fluorescence intensity also changes with probe concentration, which may be problematic in case of an uneven probe distribution. Also, the recorded probe intensity is influenced by other factors such as fluctuations in lamp intensity, photobleaching of the probe, and light scattering in the medium. Probes that respond to environmental changes by emission wavelength shifts, so-called ratiometric probes, are more desirable. The fluorescence response of such probes is more robust and reliable as the probe response no longer depends on the probe concentration and excitation light intensity and is less sensitive to the scattering of the medium. For that reason, ratiometric probes are often referred to as “self-referencing” probes. Unfortunately, ratiometric probes are far less common than intensity probes, mainly because emission wavelength shifts are generally accompanied by severe changes in emission intensity.^23−26^

Technical advancements in recent years have made fluorescence lifetime measurement an affordable, highly accessible, and user-friendly technique.^27,28^ Lifetime probes are self-referencing because lifetimes are independent of probe concentration and excitation light intensity and are hardly influenced by scattering. Using fluorescence lifetime imaging microscopy (FLIM), *spatiotemporal* probing at high spatial and temporal resolution has become an established technique.

Fluorescence intensity changes result in changes in fluorescence lifetime for most probes, as expressed in eqs 1 and 2. Therefore, intensity-sensitive probes can be used as self-referencing fluorescence lifetime probes as well. Probe requirements for lifetime probes, however, are different from those of intensity probes. The main requirements for intensity probes are high fluorescence quantum yields in the “on-state” and low quantum yields in the “off-state”, resulting in large fluorescence enhancements, FE = *I*_on_/*I*_off_. For FLIM application, probes with high fluorescence quantum yields, long lifetimes,^29,30^ and modest changes in emission intensity are required. Generally, the lifetimes and fluorescence quantum yield of probe molecules, in their bound and unbound states, are proportional, provided that the analyte binding does not severely affect the intrinsic photophysical properties of the fluorophore. For fluorophore–spacer–receptor probes, *k*_F_ will not change significantly because the binding event does not take place directly at the fluorophore. At analyte concentrations around the dissociation constant of the receptor unit, mixtures of bound and unbound probes are formed, resulting in dual lifetime emissions. While the emission intensity scales linearly with the (un)bound probe concentration, the average lifetime in this probe mixture, as given by eqs 3 and 4, does not. The fluorescence lifetime is dominated by the strongly fluorescent and long-lived species, as expressed by eq 4.^28^
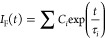
3
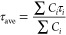
4

In eqs 3 and 4, τ_*i*_ is the fluorescence lifetime of species *i*, τ_ave_ is the average lifetime of the different probe species, and the term C_*i*_ is called the pre-exponential factor, which represents the magnitude of the species *i* in the fluorescence decay profile. It is assumed that the pre-exponential factor is the product of concentration and fluorescence quantum yield, and therefore, the lifetime response will deviate from the intensity response, in particular when analyte binding induces strong changes in fluorescence quantum yields.

The prospect of using FLIM for *spatiotemporal* probing, i.e., real-time monitoring of complex processes in three-dimensional space, has been exploited for examining biological processes, using probes that are sensitive to pH, other relevant chemical species such as reactive oxygen species,^20^ or temperature.^5^ In recent years, FLIM probes have been developed aimed at probing pH, temperatures, and chemical species under biologically relevant conditions. For pH probing, lifetime probes that monitor pH changes in mildly acidic and neutral media, in the pH domain between 5 and 8, are commercially available.^31^

Recently, real-time monitoring of complex systems in three dimensions using FLIM has also been employed for abiotic processes. Using conventional probes, pH gradients in electrochemical processes^32^ and flow-through porous catalysts have been monitored.^33^ In our current research, we are investigating electrochemical processes that are relevant for the coming energy transition, such as electrochemical water splitting and CO_2_ reduction. The electrodes in CO_2_ reduction typically operate at a high pH and produce or consume OH^–^ ions, which results in pH changes at moderately high pH values.^34^ In order to study these processes, fluorescence lifetime probes with tunable properties and sensitivities outside the biological constraints, notably for high pH values, are required.

In previous research, we have developed “switch on/switch off” fluorescent pH probes based on the 1-methyl-7-amino-quinolinium fluorophore.^14,35^ In addition, ratiometric mobility probes for monitoring physical ageing^36^ as well as determining crystallization and glass transition temperatures^37^ in amorphous and semicrystalline polymers were developed based on the same fluorophore. The 1-methyl-7-amino-quinolinium fluorophore has (photo)physical properties that are highly suitable for developing lifetime probes for FLIM applications. The fluorophore has a high fluorescence quantum yield in the 0.7–0.8 range and fluorescence lifetimes in the 12–13 ns range, which is well above the 4 ns lifetime of common fluorophores that may pollute a sample.^33^ On top of that, this fluorophore is inherently water-soluble and highly photostable. Most importantly, using the “fluorophore–spacer–receptor” configuration, the pH range in which the probes are sensitive and the extent of fluorescence quenching are easily tuned by systematic variation of the spacer–receptor units that are attached to this fluorophore.

In this research, we will demonstrate that 1-methyl-7-amino-quinolinium-based “fluorophore–spacer–receptor” probes are excellent materials for FLIM probing. The fluorescence lifetimes of these probes are pH-sensitive and easily tuned in the pH window between 5.5 and 13. FLIM measurements were demonstrated at 3 Hz with a 4 μm resolution using a 0.1 mM probe concentration. Model measurements demonstrate that pH changes due to reactions and mass transfer are visualized accurately and in great detail by FLIM measurements.

## Results and Discussion

### Probe Design and Synthesis

The general fluorescence behavior and the molecular architecture of 7-amino-1-methyl quinolinium-based pH probes with a spacer–receptor moiety attached at the 7-position are depicted in Figure 1. At low pH values, the appended amine receptor is protonated and the probe, **H**_**2**_**Q**^**2+**^ in Figure 1, is highly fluorescent with fluorescence quantum yields Φ_F_ around 0.8–0.6 and fluorescence lifetimes in the 12–13 ns domain. Upon deprotonation of the receptor, **HQ**^**+**^ is formed, and quenching by the amino functionality due to photoinduced electron transfer (PET) occurs.^22^ Both the fluorescence quantum yield and lifetime decrease. The pH value at which this transition takes place, p*K*_A1_*, depends on the excited-state acidity of the protonated amine receptor, which in turn depends on the substituents at the amino functionality, *R*_1_ and *R*_2_, see Figure 1, and the length of the spacer between the electron-deficient fluorophore and the receptor unit. In a previous contribution, we have reported p*K*_A1_^*^ values between 6 and 10,^14^ but with appropriate modifications of the molecular structure, p*K*_A1_^*^ values outside that range can be obtained in a straightforward fashion. The extent of quenching, quantified by the fluorescence enhancement FE = *I*_on_/*I*_off_, is mainly determined by the length of the spacer between the fluorophore and the receptor. The shorter the spacer, the stronger the quenching upon deprotonation is. It should be noted that changes in absorption are determined by the ground-state dissociation constant p*K*_A_, while changes in fluorescence depend on the excited-state dissociation constant p*K*_A_*, provided that equilibrium in the protonation reaction is achieved during the excited-state lifetime. For fluorophore–spacer–receptor probes, differences between p*K*_A_ and p*K*_A_^*^ values are small. Finally, at higher pH values, deprotonation of the aromatic amino proton at the quinolinium takes place and **Q**, a nonfluorescent species, is formed. This process has an apparent dissociation constant p*K*_A2_* around 11.^38^ Ground-state deprotonation is not observed for this process, not even at pH values as high as 14, which indicates that only the excited state of **Q** is acidic. Similar excited-state proton transfer (ESPT) processes have been reported for related compounds such as the “superacids” 6-,7-, and 8-hydroxyquinoline and 6-,7-, and 8-aminoquinoline compounds.^39^ The large differences in the ground-state and excited-state dissociation constants observed for these compounds are not surprising because the receptor is part of the fluorophore,^40^ which undergoes (partial) transfer of the positive charge from the quinolinium nitrogen to the amino nitrogen upon excitation.

For lifetime probing, in contrast to intensity probing, modest decreases in intensity upon receptor deprotonation are preferred. This requirement translates into the use of longer spacers (*n* = 3,4). The full-intensity quenching caused by deprotonation at the aromatic amine at high pH values around pH = 11–12 will result in lifetime changes as well. Although full-intensity quenching is often not accompanied by lifetime changes,^41^ decreases in the lifetime are expected because deprotonation takes place in the excited state by ESPT, as illustrated by Figure S10. This is explained in more detail in the Supporting Information.

**Scheme 1:**
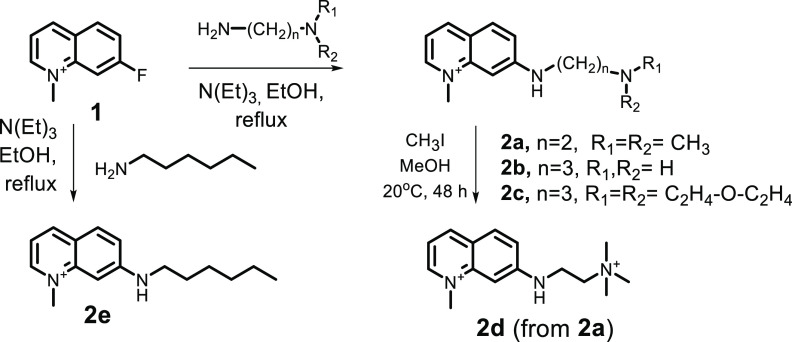
Molecular Structure and Synthesis of Probes **2a–2e**

Probe molecules **2a–2e**, depicted in Scheme 1, have been selected for further investigation. Probes **2b** and **2c** have modest 4-fold decreases in emission intensity with markedly different excited-state dissociation constants p*K*_A1_^*^ for the receptor deprotonation of 9.4 and 6.5, respectively. The fluorescence lifetimes change with a factor of 3–4 from 12.7 to 3.5 ns upon this deprotonation. The final deprotonation of the aromatic amine has a p*K*_A2_^*^ value of around 12.2 and results in full quenching of the fluorescence. Probe **2a** has a strong 80-fold decrease in emission with a p*K*_A1_^*^ value of 7.9, and due to this large quenching, **2a** is not expected to be a useful lifetime probe. Probes **2d** and **2e** do not have spacer–amino receptors attached to the quinolinium core, and fluorescence quenching takes place by deprotonation of the quinolinium amine only. For this process, a lower p*K*_A2_^*^ value is expected for probe **2d** because the ethyl trimethyl ammonium substituent at the aromatic amine in **2d** is more electron-withdrawing than the hexyl substituent in compound **2e**. Photophysical properties of probes **2a–2e**, obtained from this work and ref (14), are listed in Table 1.

**Table 1:** Photophysical Data of Probes **2a–2e** from Previous Work and Obtained in This Work

probe	**2a**	**2b**	**2c**	**2d**	**2e**
p*K*_A1_^*^(I)	7.9^b^	9.4^b^	6.5^b^		
p*K*_A1_^*^(τ)	9.3^b^	9.7^b^	6.7^b^		
p*K*_A2_^*^(I)		12.2^b^	12.2^b^	11.3^b^	11.7^b^
p*K*_A2_^*^(τ)		12.4^b^	12.4^b^	11.5^b^	11.9^b^
Φ_Fon_	0.85^a^	0.78^a^	0.74^a^	0.82^a^	0.59^b^
τ_Fon_ (ns)	13.0^b^	12.6^b^	12.7^b^	13.0^b^	11.5^b^
*I*_on_/*I*_off_	80^b^	4.1^b^	4.2^b^		
τ_on_/τ_off_		4.0^b^	3.7^b^		
λ^abs^_max_ (nm)^c^	401^b^	410^b^	410^b^	402^b^	418^b^
λ^emi^_max_ (nm)^c^	486^b^	496^b^	494^b^	484^b^	503^b^

a Taken from ref (14).

b This work.

c Maximum absorption and emission wavelength for the quinolinium in protonated form. Full absorption and emission spectra can be found in Figures S11 and S12, respectively.

### Synthesis

The fluorescent quinolinium probes **2a–2e** were synthesized by reacting primary amines with 7-fluoro-1-methylquinolinium iodide **1**(42) by a nucleophilic aromatic substitution reaction, as depicted in Scheme 1. Probes **2a**, **2c**, and **2e** were obtained by reacting **1** with a small excess of amine and were isolated in high yields after crystallization from the reaction mixtures. Probe **2b** was obtained by a similar procedure, using a 10-fold excess of diamine, in an 84% yield. Finally, probe **2d** was obtained in a 64% yield by alkylation of compound **2a** with methyl iodide in methanol at room temperature.

### Photophysical Probe Characterization

**Figure 2:**
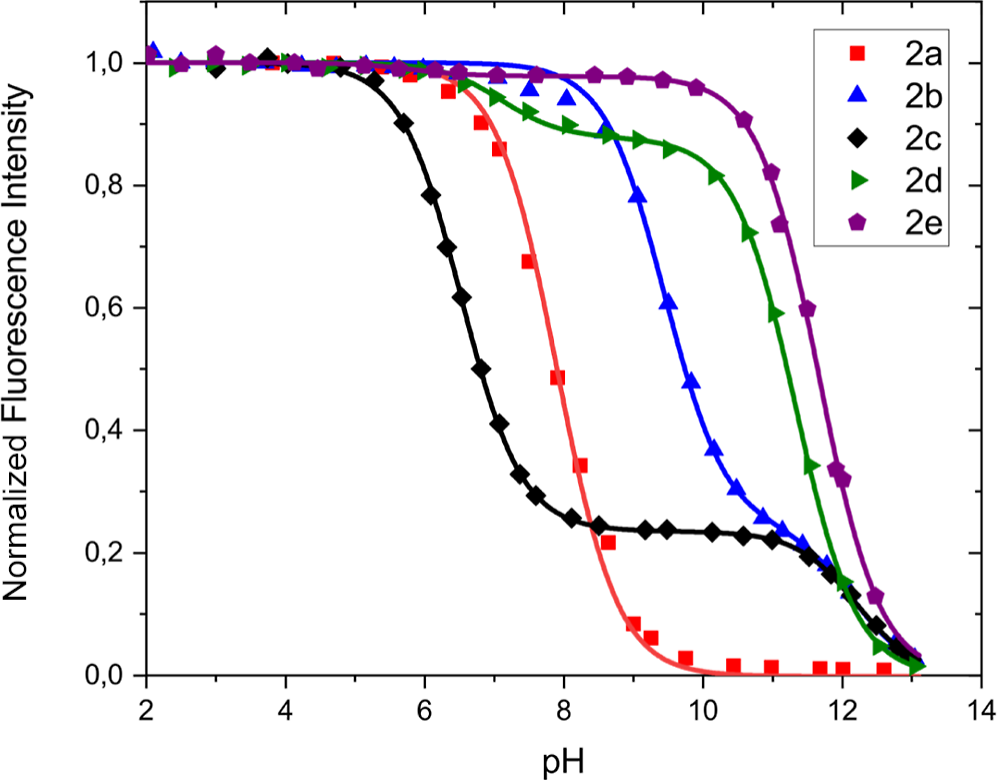
Fluorescence intensity vs pH for probes **2a–2e** in a 0.1 mM phosphate buffer. Curves connecting the data points were obtained by using eq 5 or S1.

The fluorescence emission intensities of probes **2a–2e**, as a function of pH, are plotted in Figure 2. The data points in Figure 2 are the experimental data points, the curves have been obtained using eq 5 that describes the probe composition in the excited state as a function of pH.
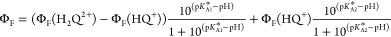
5

In eq 5, **H**_**2**_**Q**^**2+**^, **HQ**^+^, and **Q** are the protonated quinolinium probe, the quinolinium probe, and the deprotonated probe, respectively, as depicted in Figure 1. p*K*_A1_^*^ and p*K*_A2_^*^ are the excited-state dissociation constants of the **H**_**2**_**Q**^**2+**^/**HQ**^+^ and **HQ**^+^/**Q** equilibria, respectively.

From Figure 2, p*K*_A1_^*^ values of 7.9, 9.4, and 6.5 are determined for probes **2a**, **2b**, and **2c** along with fluorescence enhancements of 80, 4.1, and 4.2, respectively. These values are in good agreement with previous work.^14^ For probes **2b** and **2c**, an identical p*K*_A2_^*^ value of 12.2 was determined. Probes **2d** and **2e** do not have amine receptors appended to the quinolinium ions, so only p*K*_A2_^*^ values of 11.3 and 11.7 were observed. These p*K*_A2_^*^ values correlate very well with the electron-donating ability of the spacer–receptor unit attached to the aromatic amine at the quinolinium moiety. For probe **2d**, the strongly electron-deficient ethyl trimethylammonium unit induces the lowest p*K*_A2_^*^ value, whereas the electron-rich amine-appended propyl units in probes **2b** and **2c** induce the highest p*K*_A2_^*^ values in these compounds.

Surprisingly, decreases in emission intensity around neutral pH values were observed for compounds **2b**, **2d**, and **2e**, with the apparent p*K*_A_^*^ values close to 7.1. For compounds **2b** and **2e**, decreases in emission intensity are small, 3 and 2%, respectively, but for compound **2d**, the decrease in intensity is a more substantial 12%. We found that the origin of this decreased emission intensity is monohydrogen phosphate that is formed in the 10^–4^ M phosphate buffer around pH = 7.^43^ Similar decreases in intensity around pH 7 have been reported for phenol-appended DAOTA dyes in phosphate buffers as well.^30,44^ It was noted that this quenching increases if more concentrated buffer solutions are used, see Figures S1–S3 in the Supporting Information. We assume that it is due to hydrogen phosphate binding and that the dicationic probe **2d** has the best geometry for HPO_4_^2–^ binding. The strong quenching of probe **2d** to HPO_4_^2–^ could be interesting in the future for developing a HPO_4_^2–^ probe, similar to the anthrylpolyamines reported previously.^45^ Finally, the phosphate quenching has been incorporated in eqs S1 and S6.

**Figure 3:**
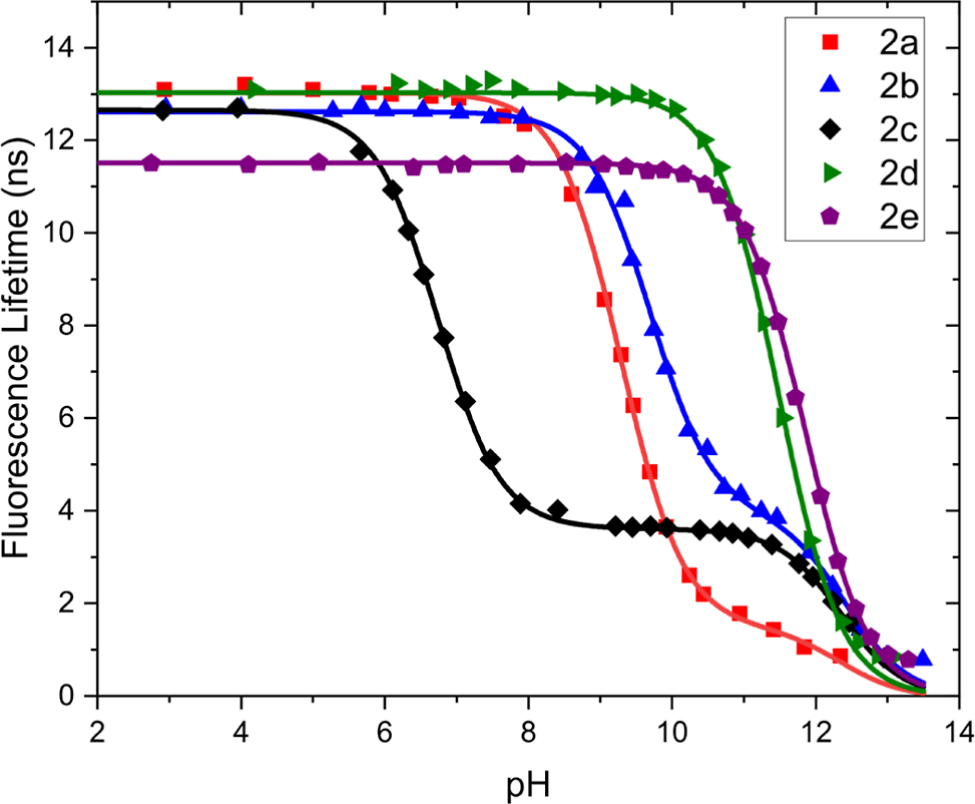
Fluorescence lifetime vs pH for probes **2a–2e** in a 0.1mM phosphate buffer. Curves connecting the data points were obtained by using eqs S5 or S6. Error bars, as included in Figures 4, 5, and Supporting Information-5, are not included for better readability.

The fluorescence lifetime of probes **2a–2e** as a function of pH is depicted in Figure 3, while normalized lifetimes versus pH plots are presented in Figure S4. At low pH values, all probes exhibit lifetimes between 11.5 and 13.1 ns, and these values are proportional to the fluorescence quantum yields Φ_F_ of these probes. Changes in lifetimes due to deprotonation of a spacer-bound amine receptor occur for probes **2a**, **2b**, and **2c** only. For probes **2b** and **2c**, by fitting the experimental data using eq S5 (see the Supporting Information), p*K*_A1_^*^ values of 9.7 and 6.7 were determined from lifetime measurements. These values were 0.2–0.3 units higher than those determined by intensity measurements. This “delayed” response in the lifetime was anticipated because mixtures of **H**_**2**_**Q**^**2+**^ and **HQ**^+^ are present in the solution, at pH values of p*K*_A1_^*^ ± 1, for which the emission is dominated by the strongly emitting, long-lived **H**_**2**_**Q**^**2+**^. For probe **2a**, due to the 80-fold quenching upon deprotonation, this effect is most pronounced and the p*K*_A1_^*^ measured in lifetime is 9.3: a 1.4 shift compared with the intensity measurements.

For probes **2b–2e**, lifetimes further decrease due to deprotonation of the aromatic amine proton. For probes **2d** and **2e**, whose fluorescence lifetime is not affected below pH 10, this decrease in lifetime is most pronounced. The p*K*_A2_^*^ values measured in lifetime are slightly higher than those measured in intensity by a value of 0.2. It should also be noted that, due to the low fluorescence intensity and short lifetimes, lifetimes reported at high pH have limited accuracy.

**Figure 4:**
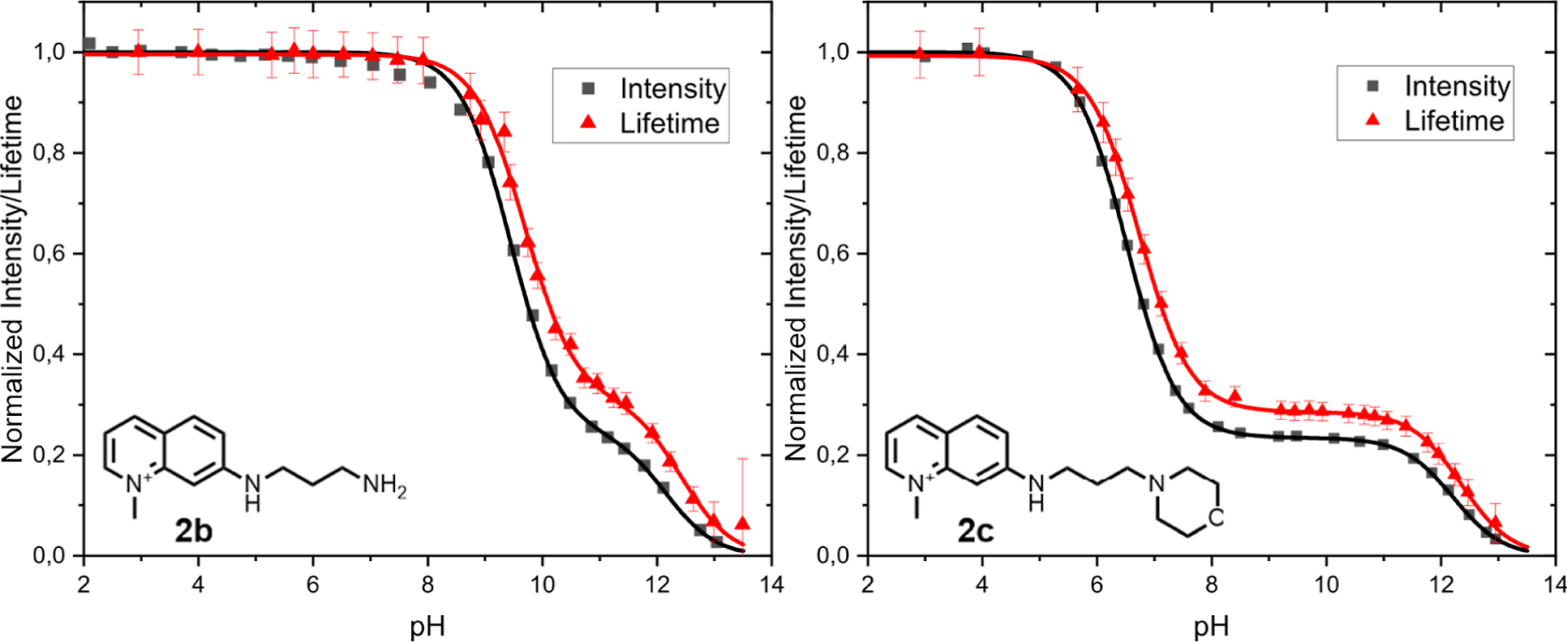
Relative intensity and lifetime vs pH of probe **2b** (left) and probe **2c** in water containing 0.1 mM phosphate buffer. The curves around the data points are generated using eq 5, S1, or S5. The error bars in this graph are the standard deviation from the FLIM measurement. See Supporting Information-6 for more information.

In Figure 4, the normalized fluorescence intensities and lifetimes as a function of pH for probes **2b** and **2c** are depicted, along with the curve fitting based on eq 5 and S5. From Figure 4, it is clear that the major differences between emission intensity and lifetime curves are that p*K*_A_^*^ values are right-shifted for the lifetime measurements and that the decreases in the lifetime upon deprotonation at higher pH values are smaller than those measured in intensity.

**Figure 5:**
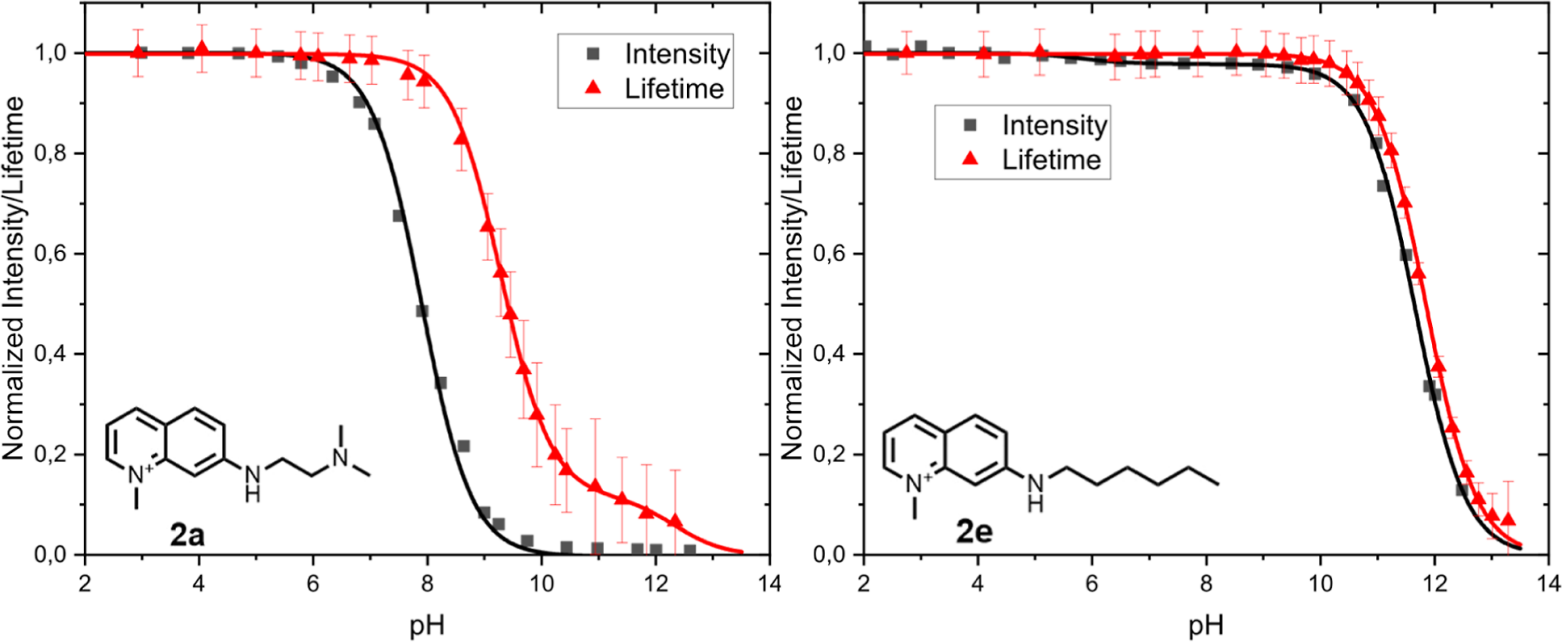
Relative intensity and lifetime vs pH of probe **2a** (left) and probe **2e** in water containing 0.1 mM phosphate buffer. The curves around the data points are generated using eq 5, S1, or S5. The error bars in this graph are the standard deviation from the FLIM measurement. See Supporting Information-6 for more information.

In Figure 5, the normalized fluorescence intensities and lifetimes as a function of pH for probes **2a** and **2e** are depicted, along with the curve fitting based on eq 5. From Figure 5, it is clearly visible that for probe **2a**, a probe that exhibits an 80-fold intensity decrease around pH = 8, the lifetime responds to pH changes at much higher pH values at which the probe emission is very low. This is so because, above pH = 8 (p*K*_A1_^*^), the lifetime is dominated by the strong emission of the protonated probe. For that reason, probes with high fluorescence enhancement, like probe **2a**, are not suitable as lifetime probes. Probe **2e** responds to pH changes only at very high values due to deprotonation of the aromatic quinolinium proton. Intensity and lifetime profiles are very similar. Figure 5b shows that, in contrast to the intensity, the lifetime of probe **2e** does not respond to hydrogen phosphate quenching and that the p*K*_A2_^*^ in the lifetime has increased by a modest 0.2. At high pH values between 11 and 12, *N*7-alkylated quinolinium probes like **2e** are very sensitive lifetime probes. A similar behavior is observed for probe **2d**, see Figure S5.

**Figure 6:**
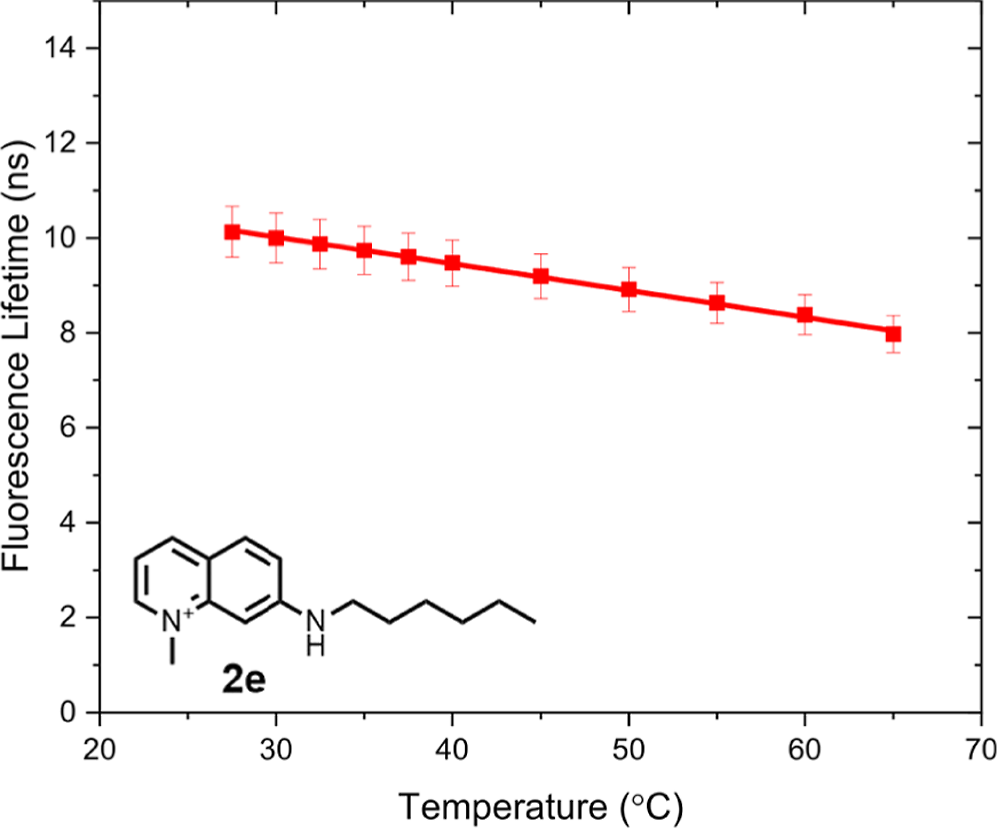
Fluorescence lifetime τ_F_ vs temperature of probe **2e** in demineralized water. The lifetime vs temperature slope is −0.057 ns/°C.

In order to be useful pH probes, the probe lifetime should not be influenced by changes in temperature and common ions, such as Na^+^, K^+^, Cl^–^, Br^–^, and SO_4_^2–^, which we expect to be present in our samples. Previous experiments with 7-ethylamino-1-methylquinolinium iodide,^14^ the ethyl analogue of probe **2e**, revealed that the probe intensity was insensitive to Cl^–^ and Br^–^ ions. In contrast, I^–^ and OH^–^ ions induced quenching due to PET and deprotonation of the aromatic amine, respectively. Similar sensitivities are expected for fluorescence lifetimes. The fluorescence lifetimes of probes **2b**, **2d**, and **2e** were investigated as a function of the temperature and the concentration of phosphate and sulfate ions. As depicted in Figures 6 and S6, S7, the fluorescence lifetime decreases upon increasing the temperature, with a gradient of 0.05–0.06 ns/^o^C. As mentioned earlier, hydrogen phosphate ions (HPO_4_^2–^) reduce both intensity and lifetime, but at concentrations below 0.1 mM, the effect is negligible for all probes in lifetime measurements and hardly visible in intensity measurements, with the notable exception of probe **2d**, see Figures S1–S3 and S8. The lifetime response of probe **2d** to the sulfate concentration is shown in Figure S9. When the sulfate concentration is increased from 10^–4^ to 10^–1^ M, lifetimes between 13.3 ns to 13.6 ns were recorded. This happens both at a pH of 7 and 3.5. These changes fall well within the standard deviation of the FLIM measurements. Hence, we can conclude that pH probing is unaffected by sulfates.

### Spatiotemporal pH Imaging in Aqueous Environments

The large pH range of the quinolinium probes enables spatiotemporal pH probing in various pH domains. To demonstrate the possibilities of the quinolinium probes, we have performed two experiments with **2b**. We probed the pH in the vicinity of a CO_2_ bubble, which dissolves in the surrounding alkaline medium, and monitored the diffusion of Ba(OH)_2_ from a small paper bag into the surrounding liquid. The FLIM setup allowed pH imaging using a 0.1 mM probe solution at a rate of up to 3 images per second and at a pixel size of 4 μm.

**Figure 7:**
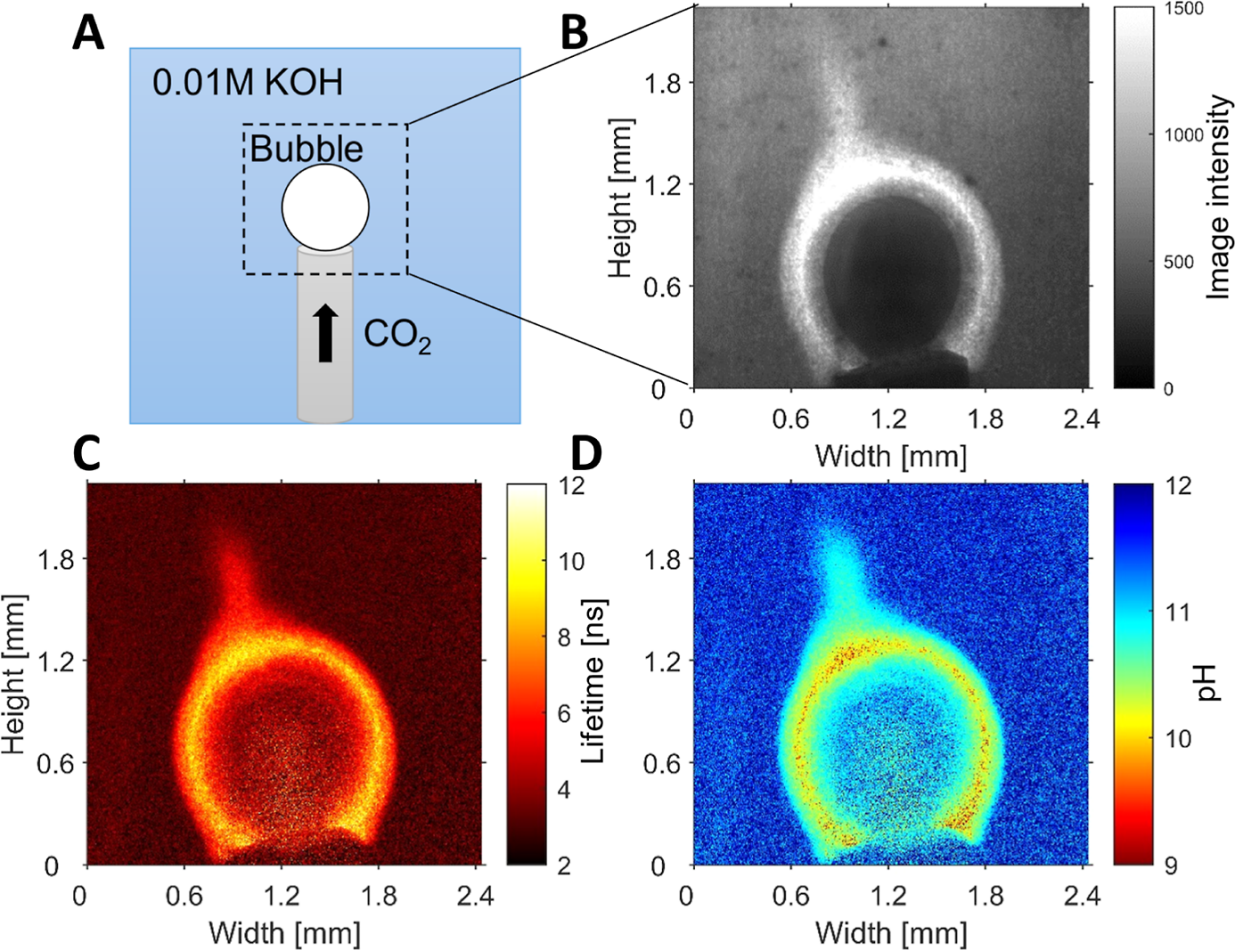
(A) Schematic representation of the experiment: A bubble of CO_2_ pumped in an alkaline (0.01 M KOH, 10^–4^ M **2b**) solution. (B) Light intensity image. (C) Fluorescence lifetime image, calculated from the phase shift from the reference. (D) pH image; lifetime image was converted using the lifetime pH curve from Figure 3.

The dissolution of gaseous CO_2_ in an alkaline medium decreases the pH value by forming carbonate ions (CO_2_ + 2OH^–^ ↔ CO_3_^2–^ + H_2_O). This phenomenon is applied when capturing CO_2_ from the air^46^ or from postcombustion gas streams.^47^ Spatiotemporal monitoring of the pH during such processes could help to gain insights into the mass transport in these CO_2_ capture processes. Figure 7 shows a bubble of CO_2_ gas which is pumped through a needle in a 0.01 M KOH solution. A higher fluorescence intensity can clearly be seen around the bubble on the intensity plot (Figure 7b). This is caused by the high fluorescence emission of the dye at lower pH, which is a result of CO_2_ dissolving and creating a locally more acidic environment close to the bubble. While the intensity images are highly dependent on dye concentration, excitation intensity, and light scattering, the fluorescence lifetime image (Figure 7c) allows us to directly measure and visualize the local pH around the CO_2_ bubble. Interestingly, both intensity and lifetime measurements show that the radial symmetry that one would expect for CO_2_ dissolution is not observed in this experiment: a lower pH is observed at the left top of the gas bubble, indicating advection in this direction.

**Figure 8:**
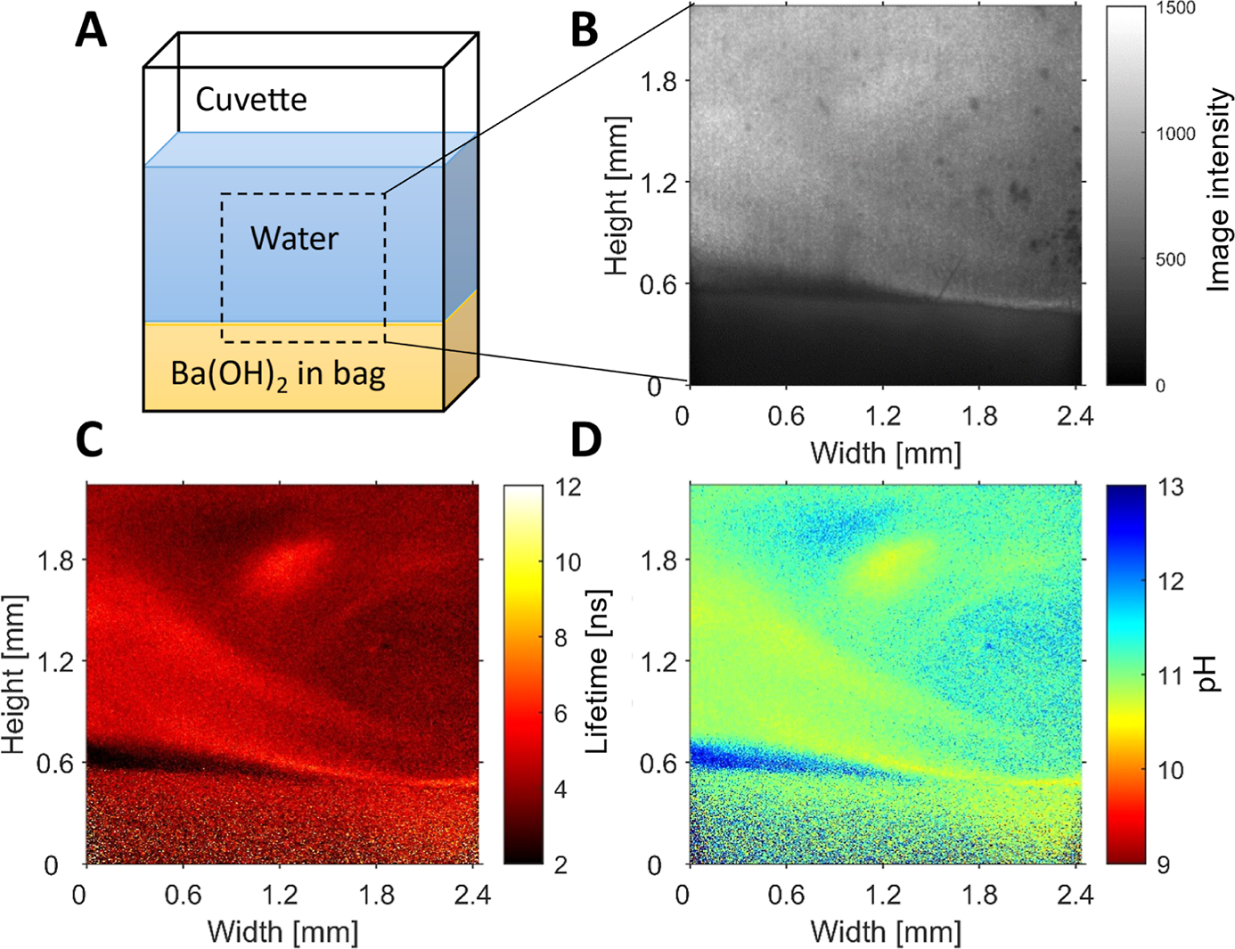
(A) Schematic representation of the experiment: Cuvette with a small paper bag filled with Ba(OH)_2_ powder, on which electrolyte (0.2 M K_2_SO_4_, 10^–4^ M **2b**) is added using a 5 mL syringe. (B) Light intensity image. (C) Fluorescence lifetime image, calculated from the phase shift from the reference. (D) pH image; lifetime image was converted using the lifetime pH curve from Figure 3.

To show that the probe works also in cases with low bulk pH and high surface pH, we injected dye solution (0.2M K_2_SO_4_, 10^–4^ M **2b**) on top of a paper bag filled with Ba(OH)_2_ and monitored the diffusion of the Ba^2+^ and OH^–^ ions into the solution (Figure 8). The lifetime and pH images in Figure 8c,d show a complex mixing behavior resulting from the liquid being injected into the cuvette and the beginning of the formation of an alkaline layer above the paper bag.

The additional strength of the FLIM technique, in combination with the quinolinium probes, is demonstrated by the pH videos in the Supporting Information. These videos allow great insights into the mixing dynamics in these systems. For example, when the CO_2_ bubble emerges from the tube, one can clearly see a swirl with a decreased pH forming above the bubble. Similar swirls were formed in repeat experiments and would be difficult to model. Also in the Ba(OH)_2_ diffusion experiment, one can see complex mixing behavior when the liquid is pumped into the cuvette and the resulting inhomogeneities in the pH of the electrolyte above the paper bag. The fast imaging of the FLIM camera, combined with the large and tunable range in pH, facilitates the mapping of mass transport in practical electrochemical flow cells, such as flow batteries or CO_2_ electrolyzers.

It should be noted that 1-methyl-7-aminoquinolinium-based fluorescent pH probes allowed for accurate pH resolution as lifetime changes are large (1–10 ns), much larger than the standard deviation in the measurements (0.5–1 ns). The intensity of the quinolinium dyes remained unchanged for long (∼1 h) experiments, which indicates high photostability. Commercial FLIM pH probes, like BCECF,^31^ SNARF-5F,^31^ and fluorescein,^48^ on the other hand, have much smaller lifetime change of around 0.5–1 ns and exhibit significant decreases in fluorescence intensity due to photobleaching under standard irradiation conditions. These commercial probes did not yield accurate and dynamic pH information in our setup and were not sensitive to high pH values. Finally, it should be noted from Figures 7 and 8 that the images taken from intensity measurements, Figures 7b and 8b, are “contaminated” with dark spots and other artifacts (a vertical line in Figure 8b) that are not present in the lifetime and pH images.

## Conclusions

In this work, we have demonstrated that 1-methyl-7-aminoquinolinium-based fluorescent pH probes are excellent materials for spatiotemporal pH probes by FLIM. These fluorescent probes are inherently water-soluble and highly photostable, have high inherent lifetimes (11.5–13 ns), and have limited sensitivity to temperature and common ions present in the aqueous media that we have investigated. Preliminary experiments have demonstrated that dynamic processes involving chemical reactions and mass transfer can be imaged with a spatial resolution of 4 μm at a rate of 3 images per second using 0.1 mM probe concentrations.

Due to the modular design, the sensitivities of the probe molecules are easily tuned in a pH range between 5 and 11 by attaching spacer–receptor units to the quinolinium core. An additional tunable regime around pH 11–13 is available by deprotonating the quinolinium fluorophore. Notably, the tunable sensitivity at high pH values is unprecedented for FLIM probes and makes the quinolinium probes excellent candidates for investigating local pH effects in complex (electro)chemical reaction systems.

Finally, it was demonstrated that the pH dependence of fluorescence lifetimes and fluorescence intensities, apart from a 0.2 pH unit shift to higher values, were very similar, for probes with modest fluorescence enhancements. Therefore, data available from pH-dependent fluorescence intensity measurements are useful input for the development of FLIM probes.

## Methods and Materials

### Probe Synthesis and Characterization

Probes **2a–2d** were synthesized according to the procedures described in ref (14). The synthesis and characterization of probe **2e** are described in the Supporting Information.

### Absorption Spectrum Characterization

The absorption spectra of the quinolinium dyes were taken on a PerkinElmer Lambda 40 by dissolving 10^–4^ M in a 10^–4^ M phosphate buffer solution in polystyrene cuvettes, one solution acidified to pH 2.5 by addition of 0.025 M HCl and one to pH 13 by addition of 0.1 M KOH.

### Fluorescence Intensity Measurements

The fluorescence intensity/pH curves were made using 200 mL of a stirred buffered dye solution, 10^–4^ M phosphoric acid, and quinolinium was added until the absorbance was 0.12 in a standard quartz cuvette. A few drops of 37% HCl were added at the start to reduce the pH to 2–3. The pH was increased by adding KOH. The pH was monitored using a 913 pH meter from Metrohm. The fluorescence spectra were taken on a Jobin Yvon-Spex Fluorolog 3–11 spectrofluorometer, and the fluorescence intensity was measured at a wavelength of the emission maximum at low pH (λ_max_).

### Fluorescence Lifetime Measurements

The lifetime/pH curves were made using 200 mL of a stirred buffered dye solution (10^–4^ M quinolinium and 10^–4^ M phosphoric acid). A few drops of HCl were added at the start to reduce the pH to 2–3. The pH was increased by adding KOH. The pH was monitored using a 913 pH meter from Metrohm (pH accuracy of ±0.003 pH), and samples were taken and stored in polystyrene cuvettes. We made sure that the total volume increase was less than 25% to prevent significant dilution of the dye.

The lifetimes were measured with a Toggel FLIM camera from Lambert Instruments (frequency domain), in combination with an X-Light V2 spinning disk confocal unit from CrestOptics (see Supporting Information-6). The solutions were excited by a 405 nm modulated laser (Omicron LuxX+ 405–300). The FLIM camera and confocal disk unit were connected to a Zeiss Axiovert 200 m microscope with a 5× objective, in which a cuvette can be mounted. The fluorescence from the samples was filtered by a long-pass filter with a cutoff at 420 nm. We used a strongly buffered solution of **2b** (pH = 8) as a fluorescence lifetime reference (τ_φ_ = 10 ns, τ_φ_ is the fluorescence lifetime calculated by phase shift) in all experiments. All reported lifetimes are calculated with the frequency method (τ_φ_) in the LIFA software from Lambert instruments. Photographs of the setup and full instrument settings are shown in the Supporting Information.

To check the validity of the lifetime measurement method, the lifetimes were also measured on a Lifespec-PS from Edinburgh Instruments (time-domain). See Supporting Information-6 for more information.

### FLIM Demonstration Experiments

The FLIM demonstration experiments were performed on the same setup as the lifetime measurements. The CO_2_ bubble dissolution experiment was done in a polystyrene cuvette (1 × 1 × 3.5 cm), and CO_2_ gas was flown through a small steel needle into the electrolyte, which consisted of 0.01 M KOH and 10^–4^ M **2b**. The Ba(OH)_2_ diffusion experiment was performed by carefully adding an electrolyte of 0.2 M K_2_SO_4_ and 10^–4^ M **2b** into a cuvette which had a small paper bag filled with Ba(OH)_2_ powder.

## Data Availability

The data supporting the findings of this study are contained within the paper and its associated Supporting Information. All other relevant data are available from the corresponding author upon reasonable request and in the Zenodo repository at 10.5281/zenodo.7802111.

## Supporting Information Available

pH vs time video of a CO_2_ bubble dissolving in the surrounding KOH liquid (AVI)
pH vs time video of BaOH diffusion from a paper bag into the surrounding liquid (AVI)
Effect of phosphate buffer, sulfates, and temperature; photophysics of HQ and Q at high pH values; absorption and fluorescence spectra; fitting the fluorescence lifetime vs pH curves; synthesis of probe 2e; and Toggel FLIM camera settings and setup (PDF)

## Supplementary Material

se3c00316_si_001.avi

se3c00316_si_002.avi
