# Supplementary material for: Quinolinium-Based Fluorescent Probes for Dynamic pH Monitoring in Aqueous Media at High pH Using Fluorescence Lifetime Imaging
Source: ACS Sens. 2023 Apr 27;8(5):2050–9. doi: 10.1021/acssensors.3c00316 (PMC10226165; doi:10.1021/acssensors.3c00316)
Supplement: Supplementary file 3 — se3c00316_si_003.pdf [file se3c00316_si_003.pdf]

Supporting Information

Quinolinium-Based Fluorescent Probes for Dynamic pH Monitoring in Aqueous Media at High pH using Fluorescence Lifetime Imaging (FLIM)

Jorrit Bleeker, Aron P. Kahn, Lorenz M. Baumgartner, Ferdinand C. Grozema, David A. Vermaas* and Wolter F. Jager*

Faculty of Applied Sciences, Department of Chemical Engineering, Delft University of Technology, 2629 HZ Delft, The Netherlands.

W.F.Jager@tudelft.nl, D.A.Vermaas@tudelft.nl

The supporting information contains following items:

SI-1. Effect of phosphate buffer, sulphates and temperature

1. Equation S1. Fluorescence intensity versus pH with phosphate buffer………Page 3
2. Figures S1-S3. Normalized intensity and lifetime of probes **2a - 2e** ....Pages 3-5
3. Figure S4. Normalized fluorescence lifetime for probes **2b**, **2d** and **2e** ….…Page 6
4. Figure S5. Normalised fluorescence intensity and lifetime of probe **2d** ……..Page 6
5. Figures S6-S7. Fluorescence lifetime versus temperature for **2b** and **2d**…..…Page 7
6. Figures S8-S9. Fluorescence lifetime versus sulphate and phosphate………..Page 8

SI-2. Photophysics of HQ and Q at high pH values.

1. Figure S10. Photo physics of **HQ** and **Q** …………………………………Page 9
2. Equations S2-S4. Photo physics of **HQ** and **Q**……………………………Pages 9-10

SI-3. Absorption and Fluorescence spectra

1. Figure S11. UV-Vis Absorption spectra of quinolinium probes 2a-2e……Page 11
2. Figure S12. Fluorescence emission spectra of quinolinium probes 2a-2e…Page 12

SI-4. Fitting the fluorescence lifetime vs pH curves

1. Equation S5. Fluorescence lifetime versus pH ……………………………..Page 13
2. Equation S6. Fluorescence lifetime versus pH with phosphate buffer……...Page 13

SI-5. Synthesis of probe 2e

1. Synthetic procedure for **2e**…………………………………………………Page 13
2. Figures S13-S16. ^{1}H and ^{13}C spectra of compound **2e** ……………………Pages 14-15

SI-6. Toggel FLIM camera settings and setup

1. Figure S17. Screenshot of the settings in the LIFA software.…………………Page 17
2. Figures S18. Example of a lifetime measurement in the LIFA software………Page 17
3. Figure S19. Photograph of the FLIM lifetime measuring setup………………Page 18
4. Figure S20. Fluorescence lifetime measured in time and frequency domain….Page 19

SI-1. Effect of phosphate buffer, sulphates and temperature

In phosphate buffers quenching of fluorescence intensity and lifetime is observed at high concentration due to the formation of hydrogen phosphate dianions (HPO_4^{2-}) at increasing pH. Phosphorous acid has 3 dissociation constants with pK_A values of pK_{A1}=2.2, pK_{A2}= 7.2 and pK_{A3}=12.4, respectively. Therefore the hydrogen phosphate quenching process will be observed around pH= 6.7.

For describing fluorescence intensity versus pH plots of probes **2a-2e**, Equation 5 is appended to form equation S1:

$$\Phi_F = (\Phi_F(H_2Q^{2+})_{H_2PO_4^-} - \Phi_F(H_2Q^{2+})_{HPO_4^{2-}})\frac{10^{(pK_{A2}-pH)}}{1+10^{(pK_{A2}-pH)}} + (\Phi_F(H_2Q^{2+})_{HPO_4^{2-}} -$$

$$\Phi_F(HQ^+)_{HPO_4^{2-}})\frac{10^{(pK_{A1*}-pH)}}{1+10^{(pK_{A1*}-pH)}} + \Phi_F(HQ^+)_{HPO_4^{2-}}\frac{10^{(pK_{A2*}-pH)}}{1+10^{(pK_{A2*}-pH)}} \quad \text{Eq. S1}$$

In Equation 5, $\mathbf{H_2Q^{2+}}$, $\mathbf{HQ^+}$ and **Q** are the protonated quinolinium probe, the quinolinium probe and the deprotonated probe, respectively, as depicted in Figure 1. $\Phi_F(H_2Q^{2+})H_2PO_4^-$, $\Phi_F(HQ^+)$ $H_2PO_4^-$ and $\Phi_F(Q)$ $H_2PO_4^-$ are the fluorescence quantum yields of $\mathbf{H_2Q^{2+}}$, $\mathbf{HQ^+}$ and **Q**, in the presence of dihydrogen phosphate anions, species that do not quench the fluorescence**.** $\Phi_F(H_2Q^{2+})HPO_4^{2-}$, $\Phi_F(HQ^+)$ HPO_4^{2-} and $\Phi_F(Q)$ HPO_4^{2-} are the fluorescence quantum yields of $\mathbf{H_2Q^{2+}}$, $\mathbf{HQ^+}$ and **Q**, in the presence of hydrogen phosphate ions and these values depend on the buffer concentration. pK_{A1}* and pK_{A2}* are the excited state dissociation constants of the $\mathbf{H_2Q^{2+}}$/ $\mathbf{HQ^+}$ and $\mathbf{HQ^+}$/**Q** equilibria, respectively and pK_{A2} is the second dissociation constant of phosphoric acid with a value around 6.7.

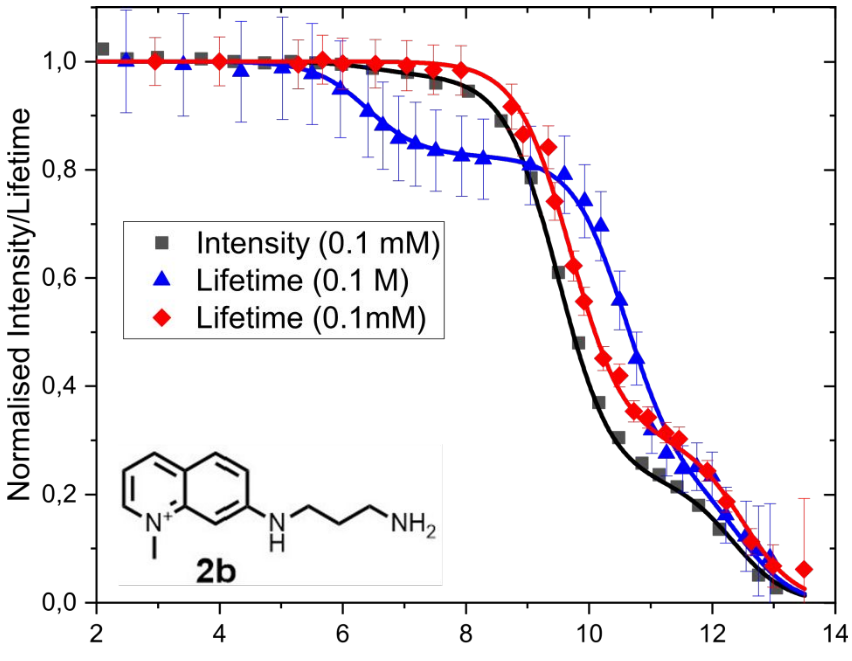

Figure S1. Normalised fluorescence intensity and lifetime of probe **2b** in 0.1 M and 0.1 mM phosphate buffer. The error bars in this graph are the standard deviation from the FLIM measurement. See SI-5 for more information.

Parameters obtained from Figure S1:

Intensity versus pH with 0.1 mM Phosphate buffer, using Eq S1:

pK_A= 6.5, pK_{A1}*= 9.4, pK_{A2}*= 12.2

($\Phi_F(H_2Q^{2+})HPO_4^{2-}$ - $\Phi_F(H_2Q^{2+})$) = 0.025, ($\Phi_F(H_2Q^{2+})HPO_4^{2-}$ - ($\Phi_F(HQ^+)HPO_4^{2-}$ = 0.75, ($\Phi_F(HQ^+)HPO_4^{2-}$ = 0.225

Lifetime versus pH with 0.1 M Phosphate buffer, using Eq S1:

pK_A= 6.4, pK_{A1}*= 10.6, pK_{A2}*= 12.5

($\Phi_F(H_2Q^{2+})HPO_4^{2-}$ - $\Phi_F(H_2Q^{2+})$) = 0.175, ($\Phi_F(H_2Q^{2+})HPO_4^{2-}$ - ($\Phi_F(HQ^+)HPO_4^{2-}$ = 0.6, ($\Phi_F(HQ^+)HPO_4^{2-}$ = 0.3

Lifetime versus pH with 0.1 mM Phosphate buffer, using Eq 5:

pK_{A1}*= 9.7, pK_{A2}*= 12.4

($\Phi_F(H_2Q^{2+})$ - ($\Phi_F(HQ^+)$= 0.7, ($\Phi_F(HQ^+)$ = 0.3

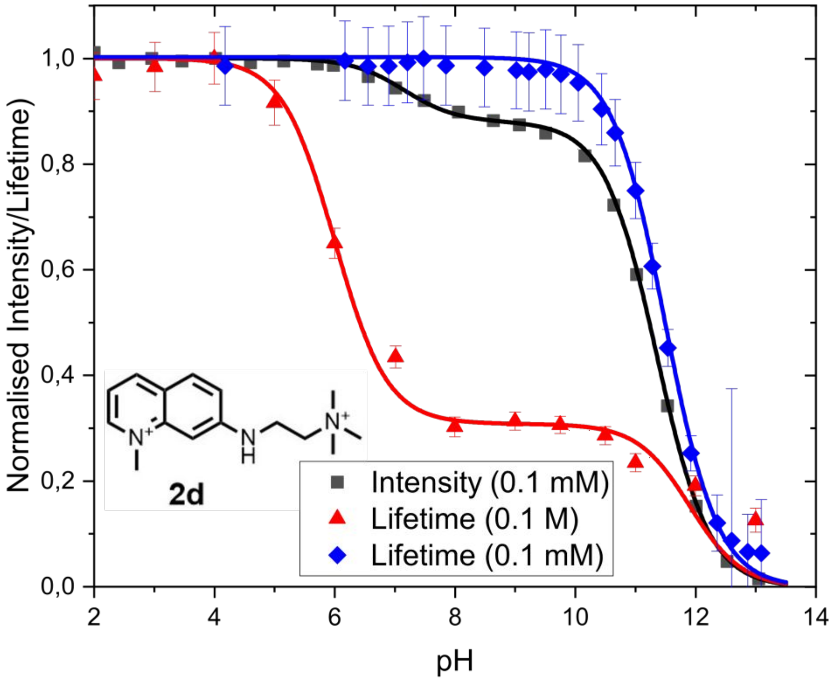

Figure S2. Normalised fluorescence intensity and lifetime of probe **2d** in 0.1 M and 0.1 mM phosphate buffer. The error bars in this graph are the standard deviation from the FLIM measurement. See SI-5 for more information.

Parameters obtained from Figure S2:

Intensity versus pH with 0.1 mM Phosphate buffer, using Eq S1:

pK_A= 7.15, pK_{A2}*= 11.3

$\Phi_F(H_2Q^{2+})HPO_4^{2-}$ - $\Phi_F(H_2Q^{2+})$ = 0.12, ($\Phi_F(HQ^+)HPO_4^{2-}$ = 0.88

Lifetime versus pH with 0.1 M Phosphate buffer, using Eq S1:

pK_A= 6.0, pK_{A2}*= 11.9

$\Phi_F(H_2Q^{2+})HPO_4^{2-}$ - $\Phi_F(H_2Q^{2+})$ = 0.69, $\Phi_F(HQ^{+})HPO_4^{2-}$ = 0.31

Lifetime versus pH with 0.1 mM Phosphate buffer, using Eq 5:

pK_{A2}*= 11.5

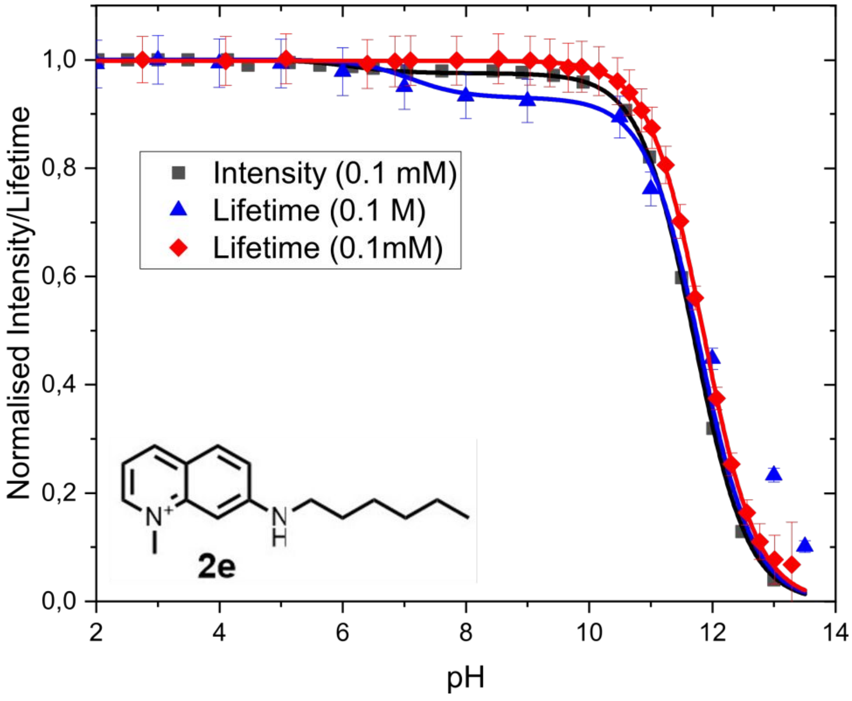

Figure S3. Normalised fluorescence intensity and lifetime of probe **2e** in 0.1 M and 0.1 mM phosphate buffer. The error bars in this graph are the standard deviation from the FLIM measurement. See SI-5 for more information.

Parameters obtained from Figure S3:

Intensity versus pH with 0.1 mM Phosphate buffer, using Eq S1:

pK_A= 6.15, pK_{A2}*= 11.7

$\Phi_F(H_2Q^{2+})HPO_4^{2-}$ - $\Phi_F(H_2Q^{2+})$ = 0.025, ($\Phi_F(HQ^{+})HPO_4^{2-}$ = 0.975

Lifetime versus pH with 0.1 M Phosphate buffer, using Eq S1:

pK_A= 7.1, pK_{A2}*= 11.8

$\Phi_F(H_2Q^{2+})HPO_4^{2-}$ - $\Phi_F(H_2Q^{2+})$ = 0.07, $\Phi_F(HQ^{+})HPO_4^{2-}$ = 0.97

Lifetime versus pH with 0.1 mM Phosphate buffer, using Eq 5:

pK_{A2}*= 11.9

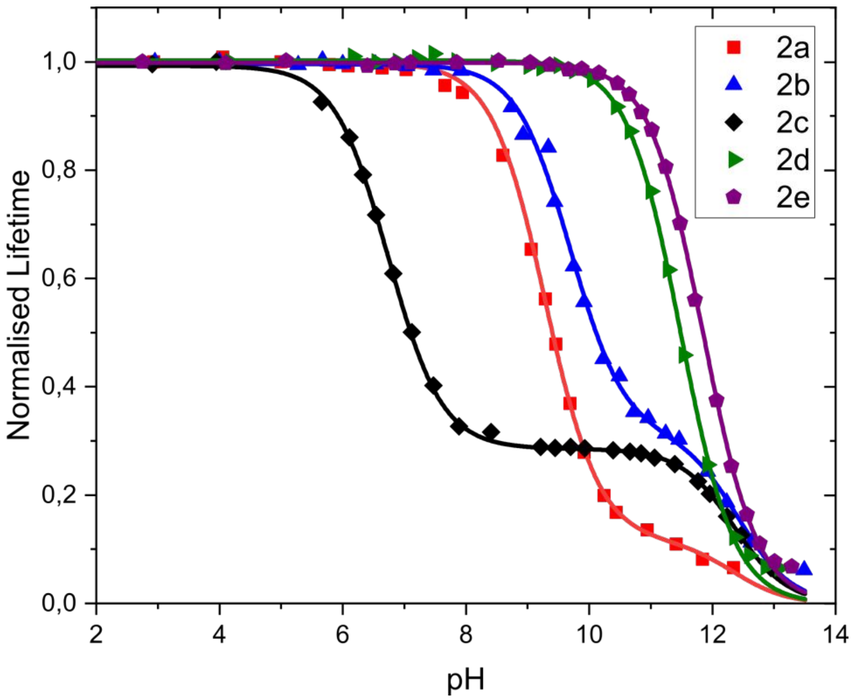

Figure S4. Normalized fluorescence lifetime vs pH for probes **2a**-**2e** in a 0.1mM Phosphate buffer. Curves connecting the data points were obtained by using Equation S5 or S6. Error bars, as included in Figures 4,5 and S1-5, are not included for better readability.

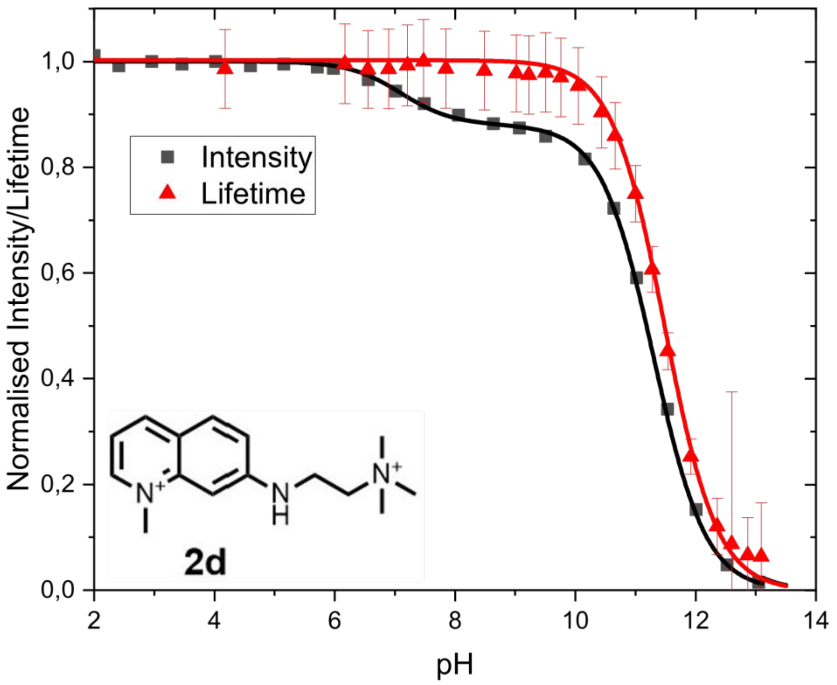

Figure S5. Normalised fluorescence intensity and lifetime of probe **2d** in water containing 0.1 mM phospate buffer. The curves connecting the data points are generated using Eq. 5 and the equation in SI-3. The error bars in this graph are the standard deviation from the FLIM measurement. See SI-5 for more information.

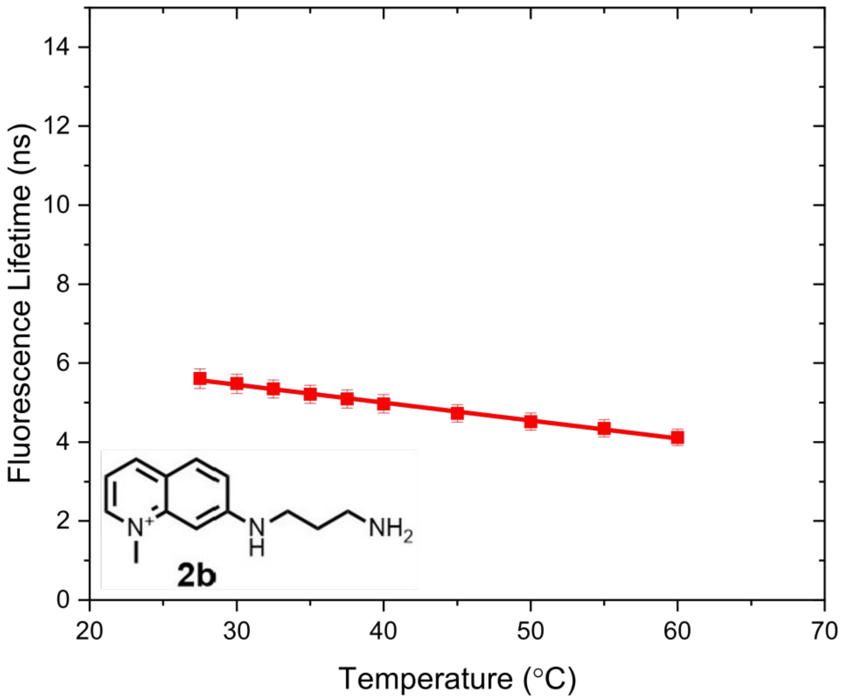

Figure S6. Fluorescence lifetime τ_F vs temperature of probe **2b** in demineralised water. The lifetime vs temperature slope is -0.045 ns/°C.

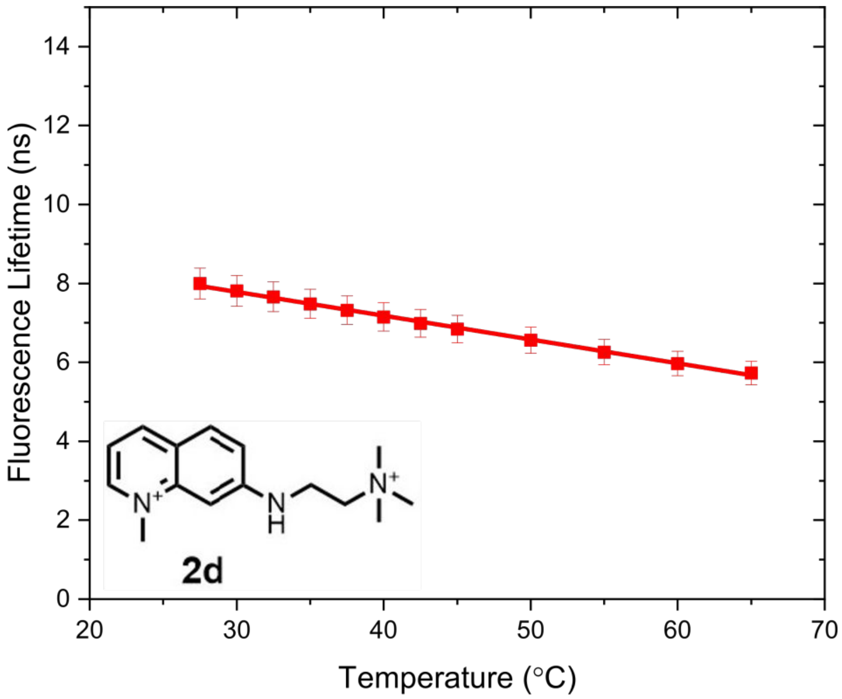

Figure S7. Fluorescence lifetime τ_F vs temperature of probe **2d** in demineralised water. The lifetime vs temperature slope is -0.060 ns/°C.

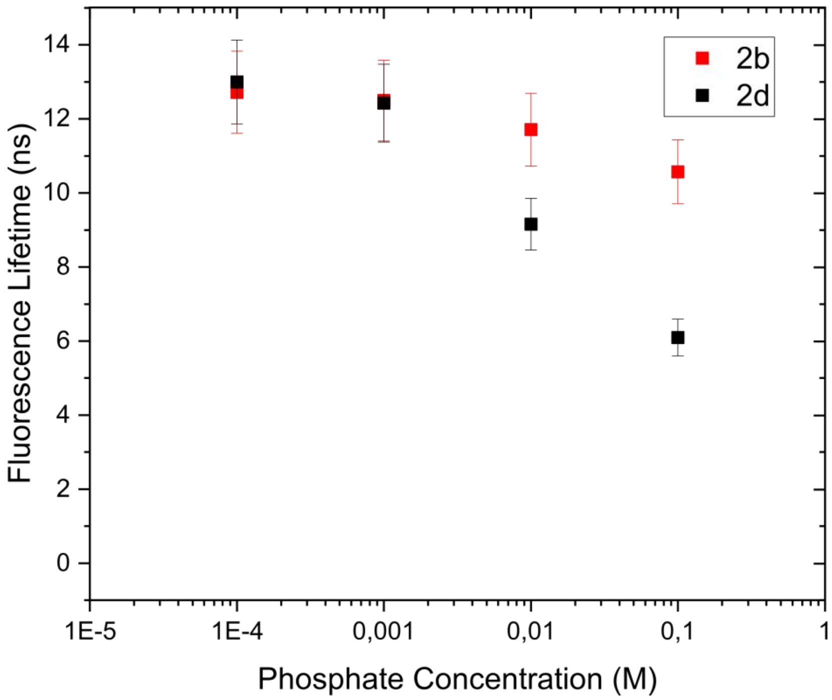

Figure S8. Phosphate dependent fluorescence lifetime of probe **2b** and **2d** in water at pH = 7.5-8.

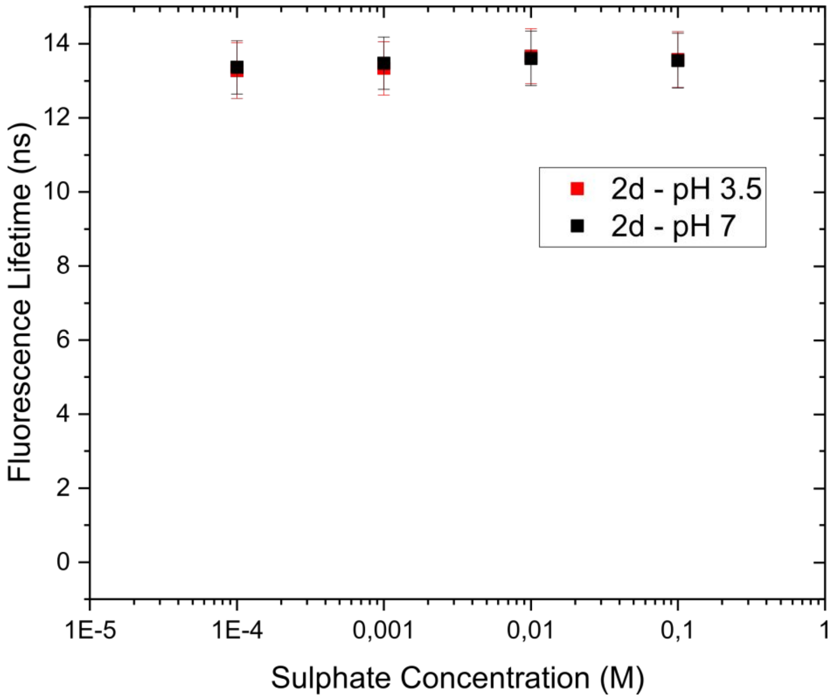

Figure S9. Sulphate dependent fluorescence lifetime of probe **2d** at pH = 7 and pH = 3.5, the solution was acidified with sulfuric acid to get the pH 3.5 solution. 10^{-4} M phosphate buffer was added to stabilize the pH.

SI-2. Photophysics of HQ and Q at high pH values.

The photophysics of alkylated quinolinium probes is depicted in Figure S10

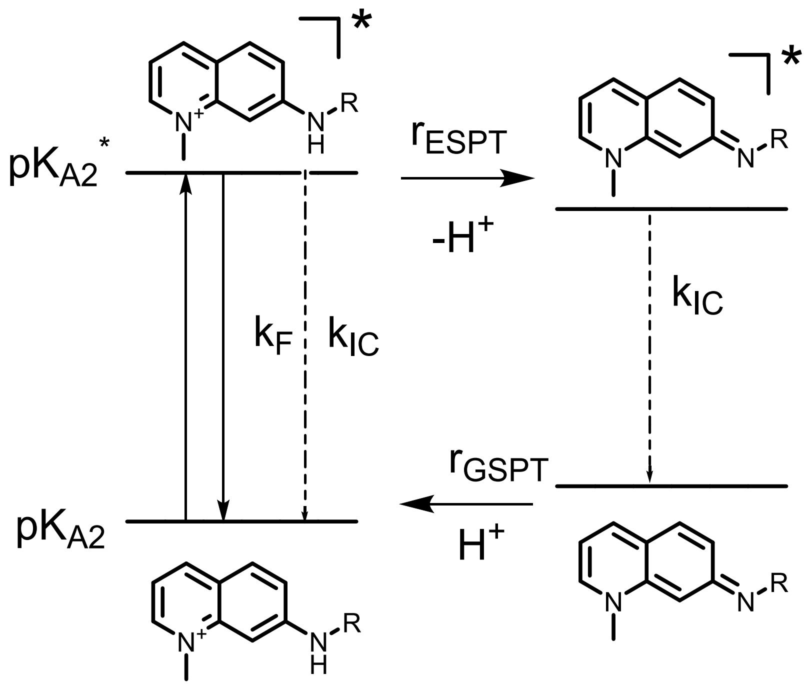

Figure S10. Photo physics of 1-methyl-7-aminoquinolinium probes at high pH values.

In Figure S10 k_F and k_{IC} are rate constants for fluorescence, non-radiative decay by internal conversion, respectively. r_{ESPT} and r_{GSPT} are the rates of excited state proton transfer and ground state proton transfer. K_{A2} and are K_{A2}* are the acid dissociation constants in the ground and the excited state, respectively.

In the ground state **HQ** is the only species that is detected by absorption spectroscopy. This is the case because pK_A has a value well above 14. In the excited state, the equilibrium composition is determined by equation S2 in which pK_{A2}* has values between 11.5 and 12.5.

$$\frac{[HQ^{+}*]}{[HQ^{+}*+Q*]}=\frac{10^{(pK_{A2}*-pH)}}{1+10^{(pK_{A2}*-pH)}} \qquad \text{Eq. S2}$$

At pH values well below pK_{A2}* only **HQ**$^{+*}$ is formed, because there is no driving force for deprotonation in the excited state according to Eq. S2. At pH values well above pK_{A2}*, non-emissive **Q*** is formed exclusively upon excitation of **HQ**$^{+}$. This is the case because the [**HQ**$^{+*}$]/[**Q***] ratio described by Eq. S2 approaches 0 and because at high pH values the rate of deprotonation r_{ESPT} (= k_{ESPT} [OH^-]) is high enough to fully deprotonate **HQ**$^{+*}$ during its lifetime. At pH values close to pK_{A2}*, mixtures compose of **HQ**$^{+*}$ and **Q*** will be formed. The ratio [**HQ**$^{+*}$]/[**Q***] will be determined by the equilibrium ratio described by Eq. S2, the rate of deprotonation r_{ESPT} and the excited state lifetime that is available to reach equilibrium. If the ESPT process is fast compared to the lifetime, i.e. r_{ESPT} >> k_{IC} ,the equilibrium constant K_A* measured from intensity versus pH plots will equal K_{A2}*. If the ESPT process proceeds slower, deprotonation lags behind and the apparent dissociation pK_A* will have a higher value.

$$I_{rel} = \frac{[HQ^{+}*]}{[HQ^{+}*+Q*]} = \frac{10^{(pK_A*-pH)}}{1+10^{(pK_A*-pH)}} \quad \text{Eq. S3}$$

When $pK_A = pK_A*$ (no ESPT), “static” mixtures of **HQ^{+*}** and **Q*** are formed intensities go down as the [**HQ^{+*}**]/[**Q***] ratio decreases. Lifetimes, however, stay constant because only **HQ^{+*}** has a finite lifetime. For that reason ESPT is a prerequisite for lifetime dependence if one of the species, **Q** in this case, is non-fluorescent.

The expression for the lifetime of **HQ^{+*}** is given in Equation S4:

$$\tau_F = \frac{k_F}{k_F + k_{ISC} + r_{ESPT}} \quad \text{Eq. S4}$$

In this Equation k_F and k_{IC}, are rate constants for fluorescence and non-radiative decay by internal conversion. r_{ESPT} is the rate of excited state proton transfer. In equation S4, r_{ESPT} is the unknown. r_{ESPT} is proportional to [OH^-] and we can write the equation $r_{ESPT=}$ k_{ESPT}[OH^-]. However, in this equation k_{ESPT} is a rate “constant” that is pH dependent, is the unknown. As is the case in the intensity measurements, a slower ESPT process will result in a higher experimental pK_A* value. In a follow-up manuscript we will address the kinetics of the ESPT process.

SI-3. Absorption and Fluorescence spectra

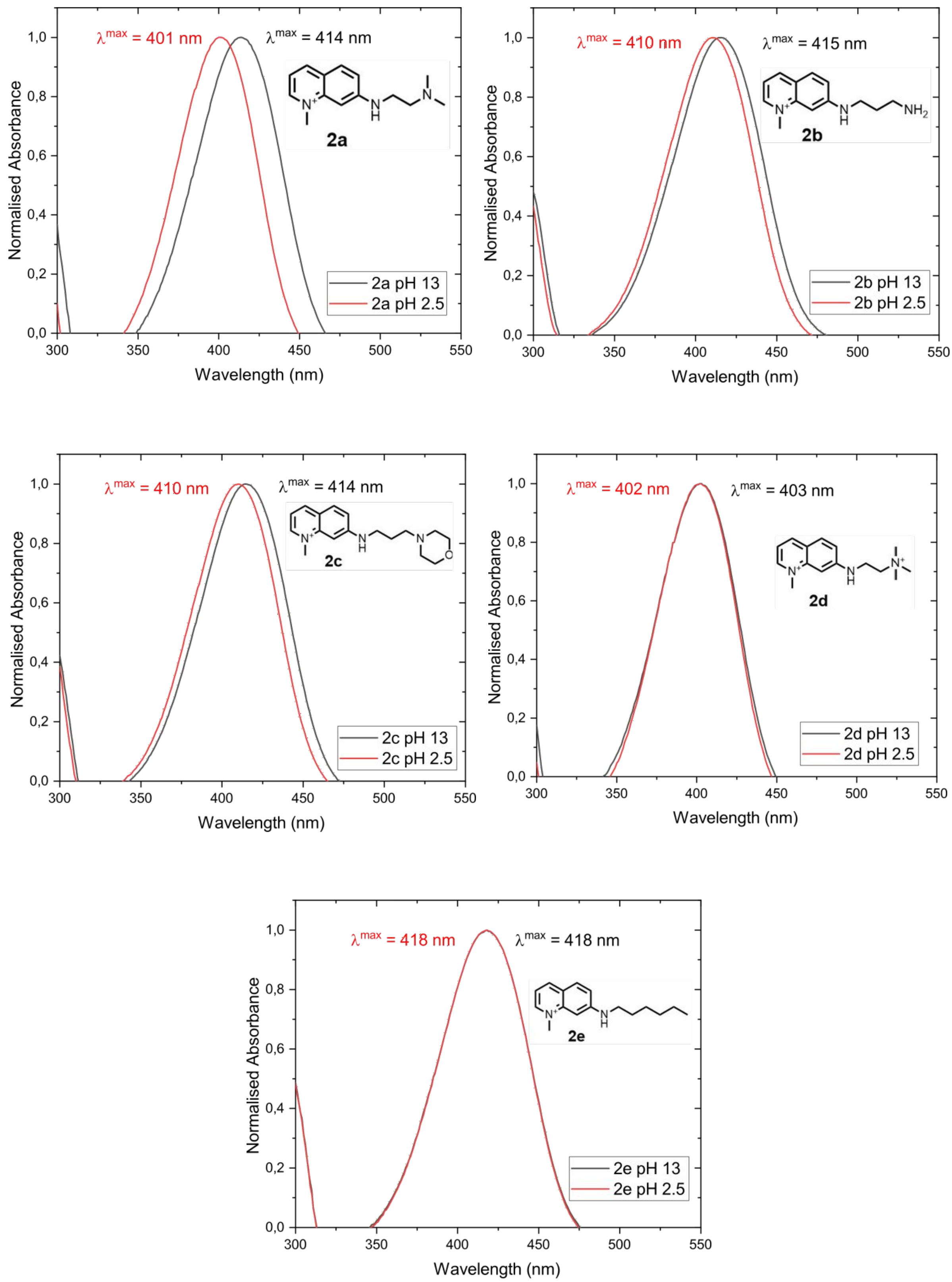

Figure S11. UV-Vis Absorption spectra of quinolinium probes **2a-2e** in 0.1mM Phosphate buffer. pH 2.5 was achieved by addition of 0.025M HCl, pH 13 was achieved by addition of 0.1 M KOH until pH was reached

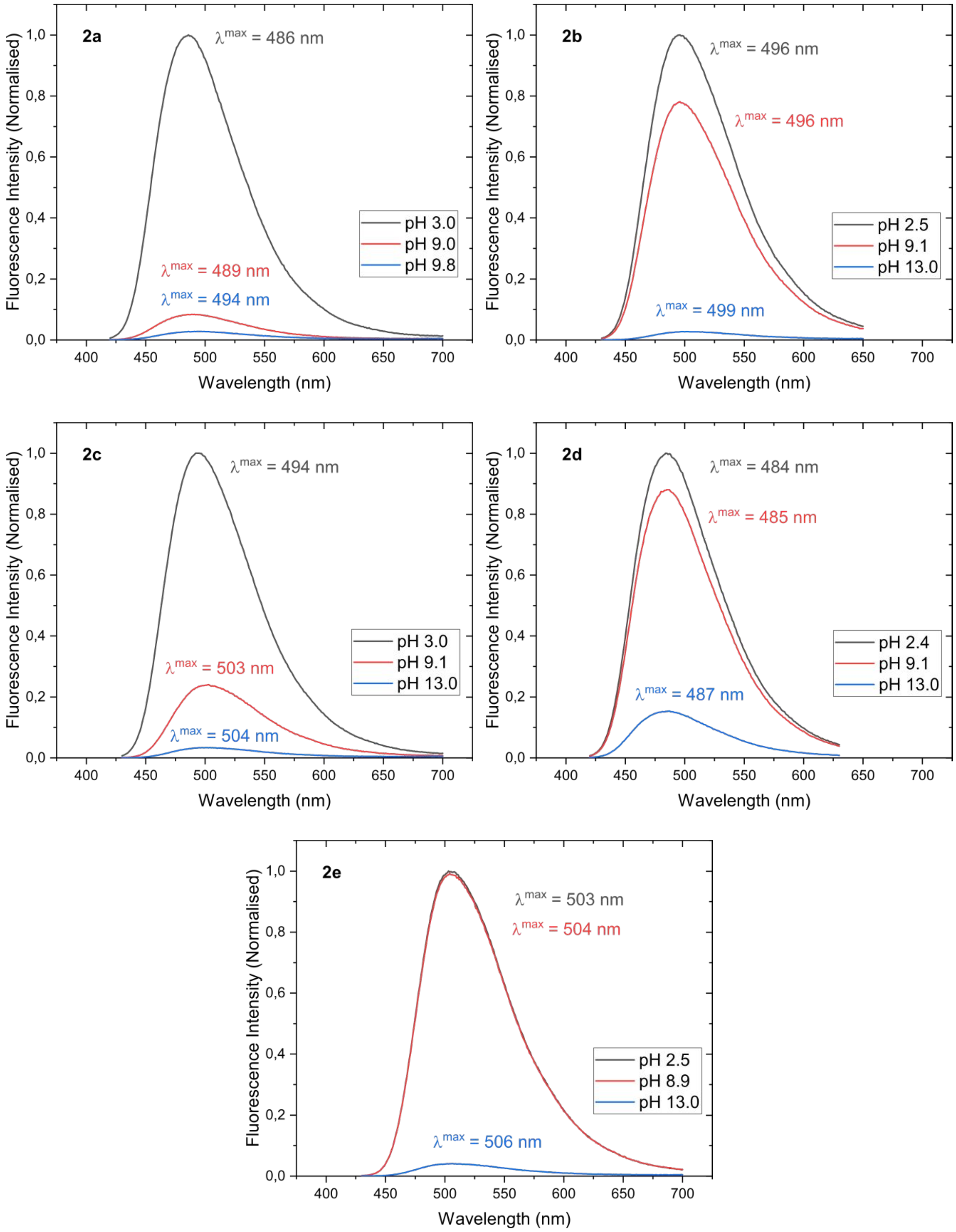

Figure S12. Fluorescence emission spectra of quinolinium probes **2a-2e** in 0.1mM Phosphate buffer

SI-4. Fitting the fluorescence lifetime vs pH curves

Fluorescence lifetime curves were fitted using Equation S5 that is similar to Equation 5, that we used for fitting fluorescence intensity curves. Equation S5 lacks a solid physical foundation, because lifetimes are proportional to the concentrations of the constituents in a mixture. Still Eq. S5 provides excellent fitting curves and the obtained pK_A* values and lifetime enhancements are reported in Table 1.

$$\tau_F = (\tau_F(H_2Q^{2+}) - \tau_F(HQ^+))\frac{10^{(pK_{A1}*-pH)}}{1+10^{(pK_{A1}*-pH)}} + \tau_F(HQ^+)\frac{10^{(pK_{A2}*-pH)}}{1+10^{(pK_{A2}*-pH)}} \quad \text{Eq. S5}$$

In case phosphate quenching occurs Equation S5 will be expanded, analogous to the expansion of Equation 5 to Equation S1 and Equation S6 is obtained:

$$\tau_F = (\Phi_F(H_2Q^{2+})_{H_2PO_4^-} - \tau_F(H_2Q^{2+})_{HPO_4^{2-}})\frac{10^{(pK_{A2}-pH)}}{1+10^{(pK_{A2}-pH)}} + (\tau_F(H_2Q^{2+})_{HPO_4^{2-}} -$$

$$\tau_F(HQ^+)_{HPO_4^{2-}})\frac{10^{(pK_{A1}*-pH)}}{1+10^{(pK_{A1}*-pH)}} + \tau_F(HQ^+)_{HPO_4^{2-}}\frac{10^{(pK_{A2}*-pH)}}{1+10^{(pK_{A2}*-pH)}} \quad \text{Eq. S6}$$

The symbols used in Eq. S6 are explained in sections **SI-3** and **SI-1**.

SI-5. Synthesis of probe 2e

7-(Hexylamino)-1-methylquinolinium Iodide (**2e**)

7-Fluoro-1-methylquinolinium iodide (1, 250 mg, 0.86 mmol), hexylamine (120 mg, 1.2 mmol), and triethylamine (95 mg, 94 mmol) in 100% ethanol (3 mL) were heated to reflux for 30 min. The reaction mixture was allowed to cool to room temperature. After addition of 1 mL of diethyl ether, orange crystals were formed and 260 mg (82%) of **2e** was obtained after filtration.

^{1}H NMR (400 MHz, DMSO-d6) δ (ppm) 8.85(1H, d, J=4 Hz), 8.65 (1H, d, J=8 Hz), 7.95 (1H, d, J=12 Hz), 7.88 (1H, t, J=4 Hz), 7.42 (1H, t, J= 8 Hz), 7.31 (1H, d, J =12 Hz), 6.67 (1H, s), 4.25 (3H, s), 3.28 overlap with H_2O (2H, t, J= 8Hz)), 1.63 (2H, m), 1.38 (2H, m), 1,29 (4H, m), 0.85 (3H, t, J=8 Hz).

^{13}C NMR (100 MHz DMSO-d6) δ (ppm) 155.0, 146.8, 144.1, 142.8, 131.6, 123.4, 122.4, 115.2, 91.0, 44.5, 43.0, 31.4, 28.2, 26.7, 22.5, 14.4.

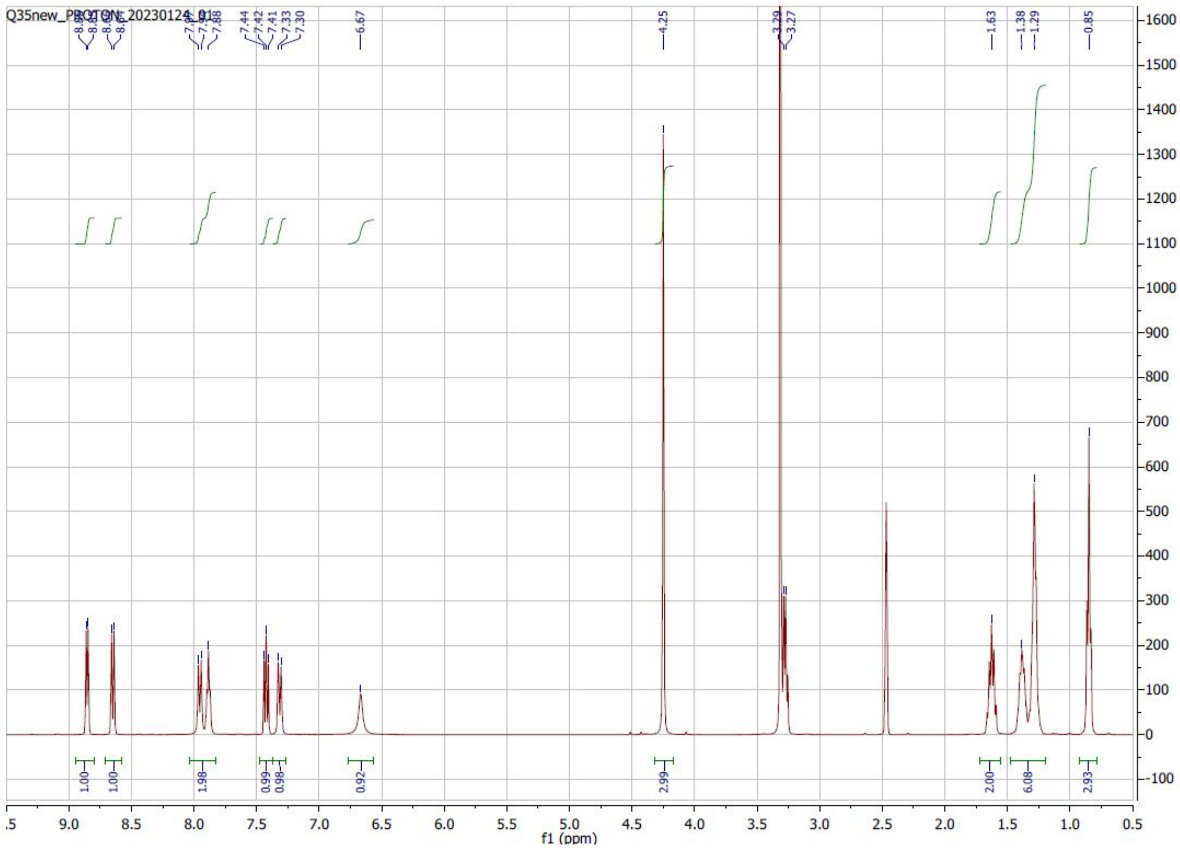

Figure S13. ^{1}H NMR spectrum of probe **2e** in DMSO-D6.

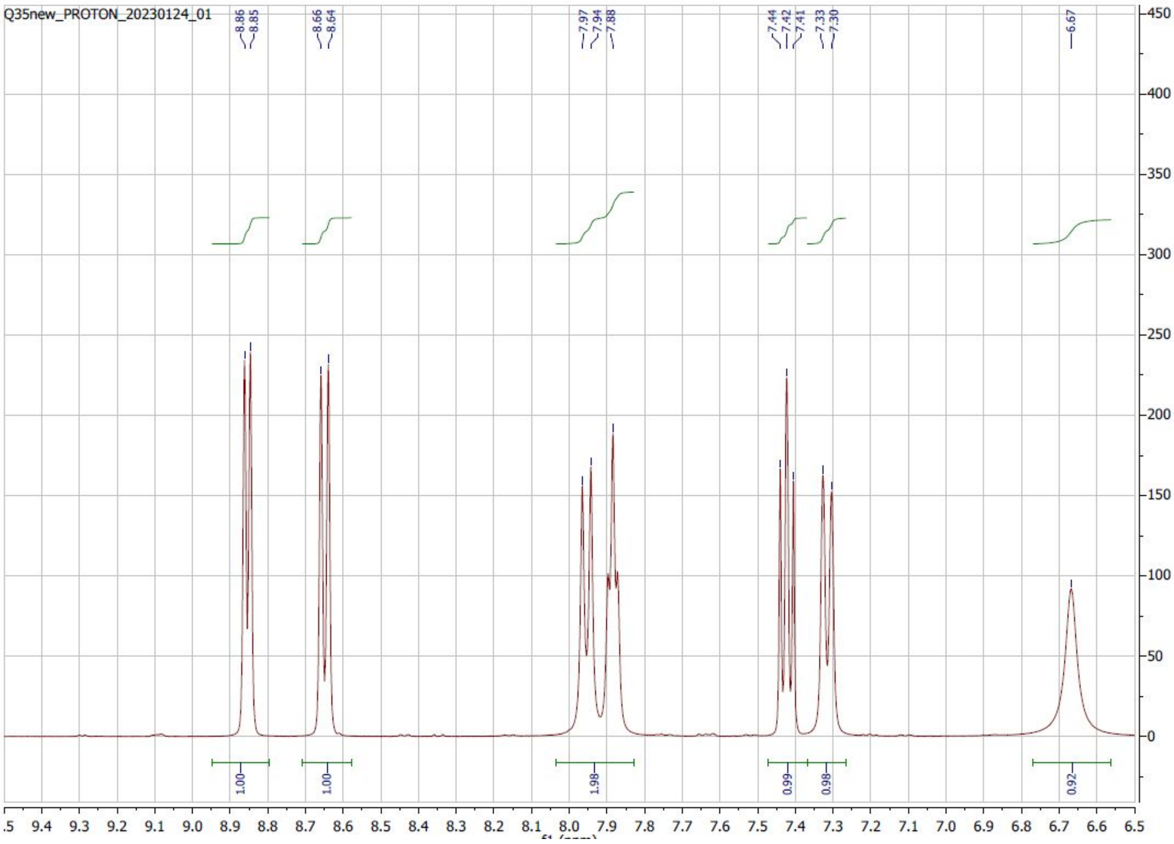

Figure S14. Aromatic part of the ^{1}H NMR spectrum of probe **2e** in DMSO-D6.

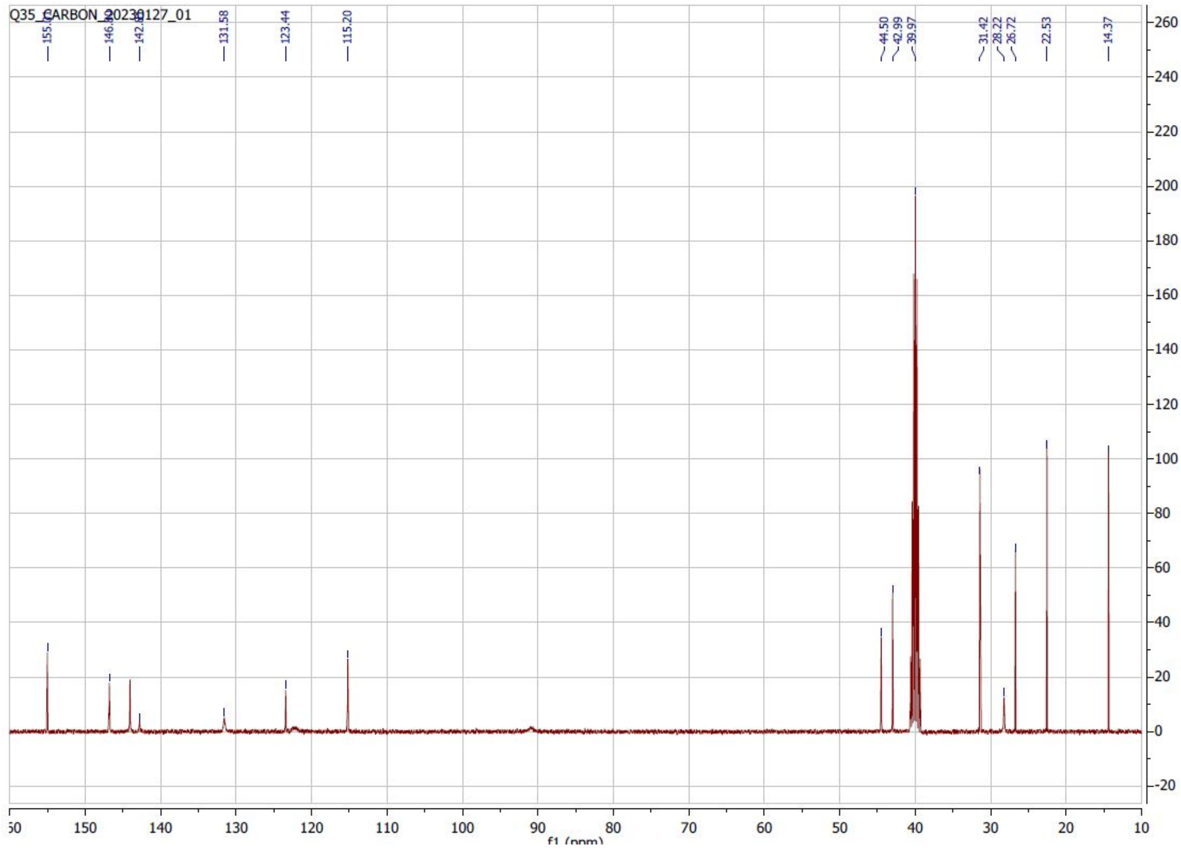

Figure S15. ^{13}C NMR spectrum of probe **2e** in DMSO-D6

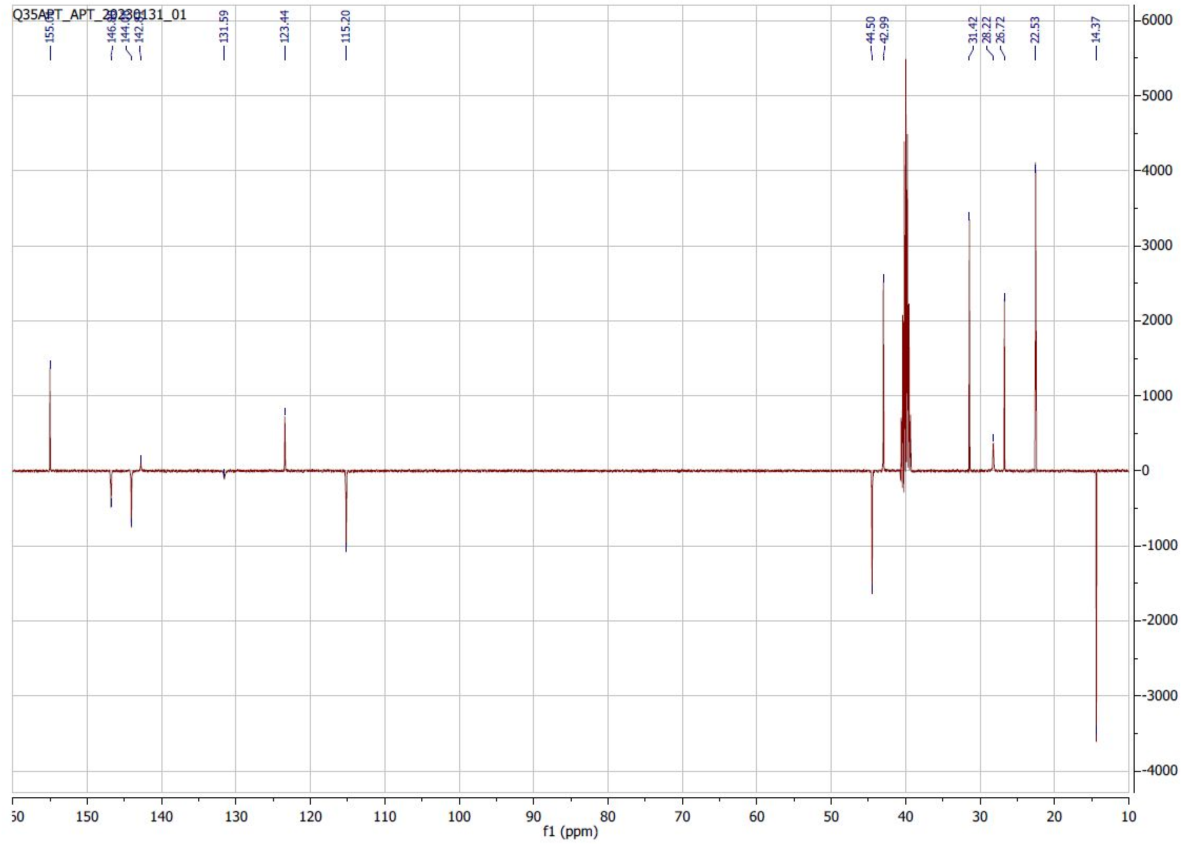

Figure S16. ^{13}C APT NMR spectrum of probe **2e** in DMSO-D6

SI-6. Toggel FLIM camera settings and setup

All lifetime measurements were performed with a Toggel FLIM camera. The solutions were placed in a cuvette and an image was taken at experimental settings shown in Figure S17. The LIFA software calculates the lifetime from the phase shift of the laser at every pixel. The lifetimes shown in the figures (i.e. Figure 3) were calculated by taking an average over all the pixels (Figure S18). This method is also known as the "Frequency domain"

Note on the error bars:

The error bars in all graphs are the standard deviations of the FLIM measurements. We acknowledge that the error bars of our measurements (in figures 4, 5, 6, S1-S7) are relatively high. There are two main reasons for this:

- At high lifetimes (>10 ns) – The FLIM camera calculates the lifetime at >200000 pixels and takes an average over all these pixels to calculate the average lifetime (Figure S18). However, exponential decay is a random process and at every pixel the lifetime can therefore be slightly different. At larger lifetimes the excited state molecule is more stable and therefore results in a larger spread of times (which in turn results in a larger standard deviation).
  Also, in all measurements the modulation frequency was 20 MHz, which is optimal for detecting lifetimes of around 8 ns. The higher lifetimes (12 ns+) would be more accurate if a modulation frequency of ~15 MHz was used.
- At low light intensities – At low light intensities we also observed large standard deviation, this is mainly due to the poor signal to noise ratio at these conditions.
  The noise in the measurements was usually around 50-100 counts, which was similar to the signal at the lower (1%) fluorescence intensities.

The setup is shown in Figure S19.

A more standard method of determining the lifetime of a fluorescent probe is by looking at the exponential decay of the light intensity, which is also known as the "Time domain".

In order to translate lifetimes to pH values reliably, we have compared lifetimes measured in the time domain with those measured in the frequency domain in our FLIM microscope setup. The data collected for probe **2b**, presented in Figure S20, clearly demonstrate that lifetimes determined by both methods are identical.

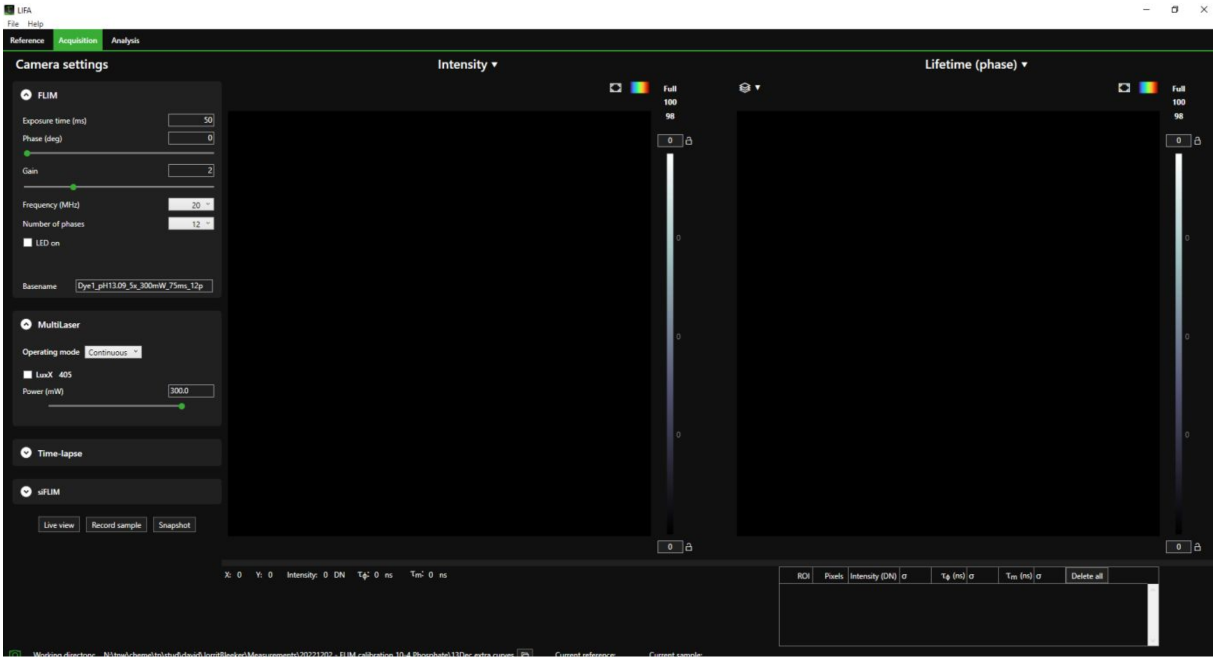

Figure S17. Screenshot of the settings in the LIFA software. The experiments were all performed at maximum laser power (300 mW), 20 MHz laser modulation, 50 ms exposure time, an image intensifier gain of 2, and 12 lifetime phases of the frequency.

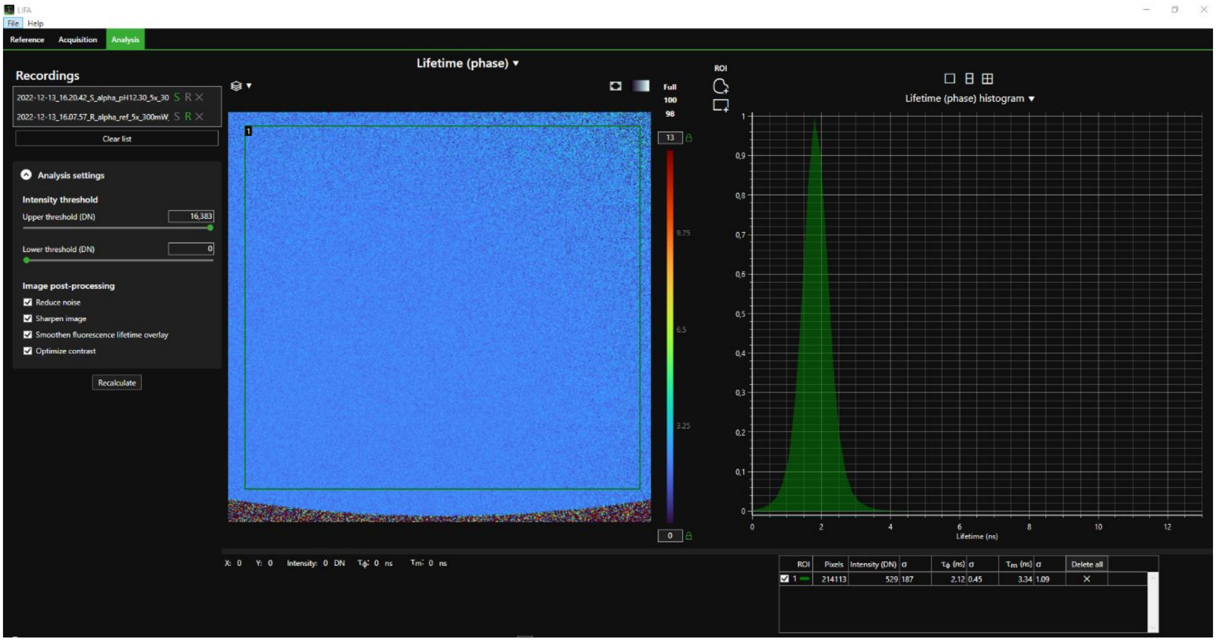

Figure S18. Example of a lifetime measurement in the LIFA software. The measured lifetime is an average of the majority of the pixels in the screen.

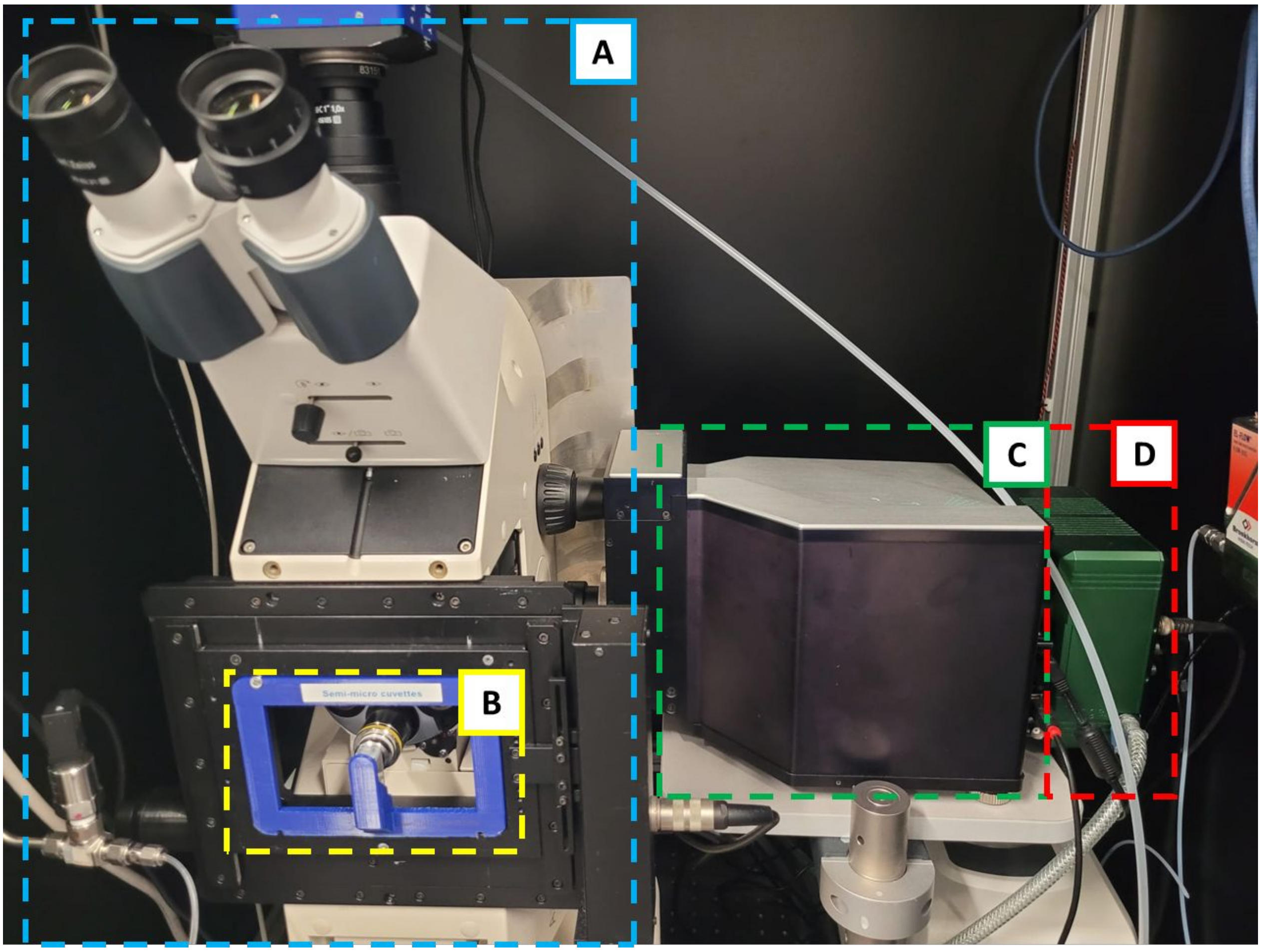

Figure S19. Photograph of the FLIM lifetime measuring setup. **A.** Zeiss Axiovert 200m Microscope to which everything is attached **B.** Objective and 3D printed sample holder **C.** CrestOptics X-Light V2 spinning disk confocal unit, the 405 nm laser is connected at the back **D.** Lambert Instruments Toggel FLIM camera.

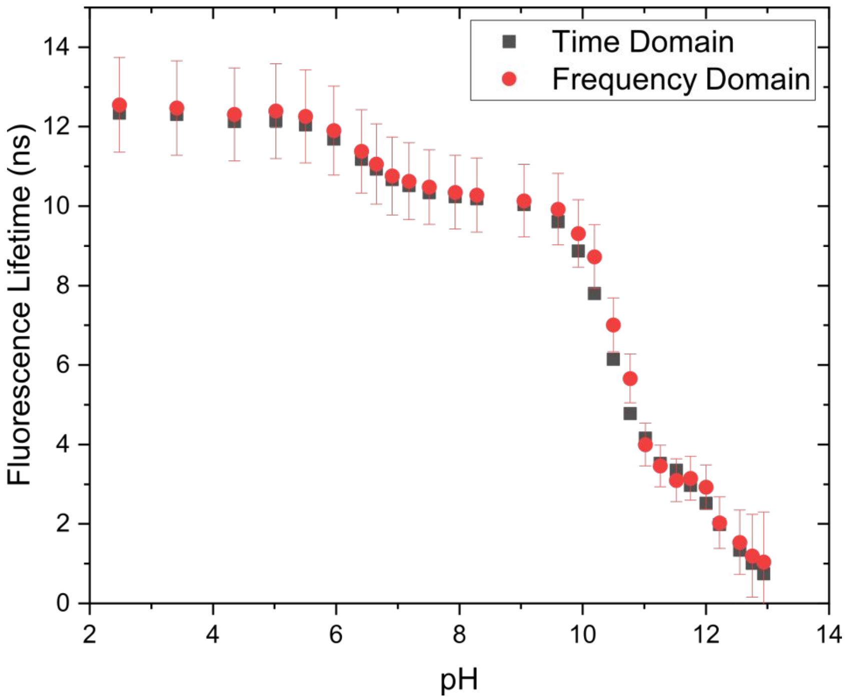

Figure S20. Comparison of fluorescence lifetime of probe **2b** in a 0.1 M phosphate buffer as a function of pH measured using time domain (black squares) or frequency domain (red circles) measurements.
